# Wind turbine database for intelligent operation and maintenance strategies

**DOI:** 10.1038/s41597-024-03067-9

**Published:** 2024-02-29

**Authors:** Pere Marti-Puig, Alejandro Blanco-M., Jordi Cusidó, Jordi Solé-Casals

**Affiliations:** 1 Data and Signal Processing Research Group, University of Vic–Central University of Catalonia, 08500 Vic, Catalonia Spain; 2 Enginyeria de Projectes i de la Construcció EPC, Polytechnic University of Catalonia, 08028 Barcelona, Catalonia Spain

**Keywords:** Wind energy, Energy management, Climate change

## Abstract

With the aim of helping researchers to develop intelligent operation and maintenance strategies, in this manuscript, an extensive 3-years Supervisory Control and Data Acquisition database of five Fuhrländer FL2500 2.5 MW wind turbines is presented. The database contains 312 analogous variables recorded at 5-minute intervals, from 78 different sensors. The reported values for each sensor are minimum, maximum, mean, and standard deviation. The database also contains the alarm events, indicating the system and subsystem and a small description. Finally, a set of functions to download specific subsets of the whole database is freely available in Matlab, R, and Python. To demonstrate the usefulness of this database, an illustrative example is given. In this example, different gearbox variables are selected to estimate a target variable to detect whether or not the estimate differs from the actual value provided for the sensor. By using this normality modelling approach, it is possible to detect rotor malfunction when the estimate differs from the actual measured value.

## Background & Summary

Wind energy is essential for meeting the EU Commission’s ambitious climate and energy targets for 2020, which include generating at least 20% of electricity from sustainable sources^1^. However, the operation and maintenance (O&M) costs of wind farms, which range from 10% to 35% of overall generation costs, pose a challenge^2^. By reducing these costs, wind farms can become more competitive with fossil fuels and expedite the transition to sustainable energy^3^.

To ensure effective management of wind farms (WF), wind turbines (WT) are scheduled for preventive maintenance every 2500 to 5000 hours. However, relying solely on preventive maintenance is insufficient to detect and predict device conditions and anticipate potential failures. The unexpected shutdown of turbines incurs substantial costs, especially considering the logistical challenges of remote locations and the time required for component replacement and on-site repairs. Preventive maintenance schedules for wind turbines are insufficient to detect and predict device conditions and anticipate failures. The life expectancy of WT is commonly estimated at around 20 years, and on average one week of downtime per year is required due to maintenance^4^. This is particularly relevant for those turbines that have been installed in the 1990s and early 2000s that are approaching the end of their lifetime. WF operators have adopted a wide range of measures to extend the operative time of their assets, as mentioned in^5^. Identifying the root causes of failures leading to turbine downtime is essential in reducing inactivity and promptly addressing critical failures. Adopting effective methodologies and tools that assist in this process can significantly benefit wind farm owners by increasing energy production, availability, and cost savings.

### Phisycal and data-based modelling

The early deterioration of WT’s systems and subsystems can be detected using their physical models or building models from their generated data. The physical model approach is useful for determining and capturing how the various components of turbines work. Monitored components are modelled into systems of physical equations that describe their behaviour from a thermodynamic, electrical, or mechanical perspective. Building physical models of wind turbines requires deep knowledge and expertise in operation principles and a deep knowledge of the WT components. Sometimes, even WT owners do not have all the required information, as manufacturers do not always share the details of the turbine’s inner systems. Nonetheless, such models have been presented in various studies in the literature. For instance, in^6^, a physical simulation of the loads of a turbine gearbox is proposed, showing that it can determine the effect that varying loads have on the component’s lifespan. Such work requires a dynamic study of the gear conditions and Finite Element Method simulations. The works presented in^7,8^ attempt a different approach by first determining the thermal network that describes gearbox conditions. Overall, physical models are a valid option for better understanding the inner workings of turbine components and generating new knowledge about them. Physical models are far more reliable than data-based ones when cause-effect relationships must be determined. However, the demanding data requirements, the availability of the necessary design parameters, and the scarce reusability of the wind turbine physical models are why the industry requires different and more flexible tools.

The main alternative to physical models are data-based models, which have risen in popularity thanks to the advancements in machine learning and statistical modelling. Only minor assumptions of the systems under analysis are needed, as the physical relations governing the operation of the various components are inferred from the data. Data sources available to study WT’s behaviour include dedicated sensors that record vibrations and acoustic emissions in mechanical components, such as the gearbox and bearings of the turbine transmission^9,10^. For electrical components, current signatures can be analyzed^11^. However, these three options are particularly expensive as these sensors are not part of the standard equipment of wind turbines. Moreover, installing additional sensors poses a logistic challenge as operations need to be halted.

Wind farms can be of different sizes, ranging from small farms with a few turbines to huge farms with hundreds or thousands of turbines. On the other hand, all wind farms have different behaviours, depending on their geographical location, wind conditions, etc. Large wind farms can also have sectors with different behaviours due to their large geographical extension and the effect that the same turbines exert on each other. Many of the wind data sets currently in use are not publicly available, which challenges the reproducibility of research, particularly in commercially important areas such as wind energy forecasting. Some databases are from offshore WFs, such as^12,13^. An interesting overview of open-source wind energy data is available in^14^. Other databases include aggregated data, which lack turbine-level measurements and turbine-specific energy production. Instead, they comprise aggregated wind energy data covering various spatial scales, from wind farms to entire countries. These datasets differ from turbine-level data in their lower temporal resolution, consisting predominantly of hourly data. Our database is unique because it provides all the raw data of the system for all the WTs and a long period of time.

### Condition monitoring via supervisory control and data acquisition system

Condition Monitoring Systems (CMS) employ various strategies, including machine learning techniques such as Artificial Neural Networks (ANN) and Self Organizing Maps (SOM), to analyse wind turbine data and identify deviations from normal behaviour^15^. These methodologies utilize SCADA data for condition monitoring, enabling the prediction of turbine failures. However, classification models in this context pose challenges due to imbalanced data distributions, where most examples belong to the healthy state, and only a few represent the alarm state. Furthermore, the accuracy of these models is hindered by labelling errors in the data. A more efficient maintenance approach known as condition-based maintenance (CBM) has emerged to address these issues. CBM involves ongoing surveillance and the detection of emerging faults through CMS, which acquires and pre-processes sensor data, evaluates it, and interprets the results^16^. CBM enables early detection of incipient faults and proactive planning of maintenance tasks, thus optimizing wind turbines’ operation and maintenance process. The literature related to WT maintenance is rich in complex attempts to anticipate failures, ranging from signal processing analyses to physical simulations and machine learning algorithms^17,18^. Researchers often neglect the scalability of solutions, likely due to a lack of large-scale datasets, including multiple wind farms and manufacturers. Most research is developed for individual wind farms or using laboratory simulations. Rarely are algorithms tested on multiple sites characterized by various turbine technologies and environmental conditions. This is a crucial shortcoming of the literature this dataset aims to address.

A valuable data source is the utilization of the Supervisory Control and Data Acquisition System (SCADA), a network of sensors monitoring the status of the turbine. SCADA data was initially designed to provide information to verify the correct operation of turbines and not as a means to assess the health status of individual subsystems. The number of sensors monitored by SCADA can vary between turbine manufacturers, though in general, the major components of the turbine are all instrumented. The resolution of SCADA data can vary, but most commonly is 5–10 minutes, and it is of higher frequency only on very rare occasions. Physical quantities such as temperatures, speeds, pressures, and states of the turbine are included in a SCADA dataset. One valuable characteristic of SCADA data is that it is available and standardized for most turbines, meaning that algorithms for its analysis are more easily transferable from one manufacturer to another. Moreover, being part of the standard instrumentation, it does not require additional investments by the wind farm owner. The importance of SCADA data for predictive maintenance and monitoring has greatly increased in the last decade. The works in^19,20^ are two of the first attempts to use SCADA data for WT condition monitoring. The methods to analyze and extract information have greatly improved from the early days. In the literature, algorithms are available to assess the health of all major components using diverse approaches based on statistical analyses, machine learning, and deep learning^17,18^. The benefits of SCADA are its wide availability, highly standardized format, and low cost. Nonetheless, its low data acquisition rate has been mentioned as an important limitation that can hinder the capability of correctly modelling the status of a turbine and detecting failures^21^.

The use of machine learning, normality models or other types of modelling strategies based on data analysis can be used for O&M. The illustrative example presented in this manuscript highlights how extreme learning machines (ELMs) can be used to predict a variable from other variables in the system, which can help to detect a malfunction of the wind turbine (specifically the gearbox in the provided example), and hence the deployment of a maintenance check of the wind turbine. To advance the development of condition-based maintenance (CBM) strategies, we release a comprehensive 3-year dataset. This dataset covers all the information obtained from the SCADA system of a wind farm, which includes five 2.5 MW Fuhrländer FL2500 wind turbines. The interesting fact about the database is that it is complete, containing all the information from the wind farm’s SCADA system. No variable or information has been modified, and therefore it can be a good starting point for experimenting with this type of data and its use to improve O&M strategies.

This dataset, already used in other papers (see^22–24^ as examples) is presented in raw, unprocessed form, as supplied by the system. It includes all variables and alarms from the different systems and subsystems of the WTs.

## Methods

The dataset contains 312 analogous variables recorded at 5-minute intervals by the wind farm’s SCADA, from 78 different sensors. Wind turbines consist of nine main systems, namely Converter, Generator, Nacelle, Rotor, Tower, Transformer, Transmission, Turbine, and Yaw. Some of these systems are further divided into specific subsystems. Specifically, there are 16 identified subsystems including Hub, Hydraulic System, Main Bearing, Pitch, Power Cabinet, Roof, Rotor, Tower, Transformer, and Yaw. For a detailed overview of the relationship between the systems and their corresponding subsystems, please refer to Supplementary Table 1.

**Fig. 1:**
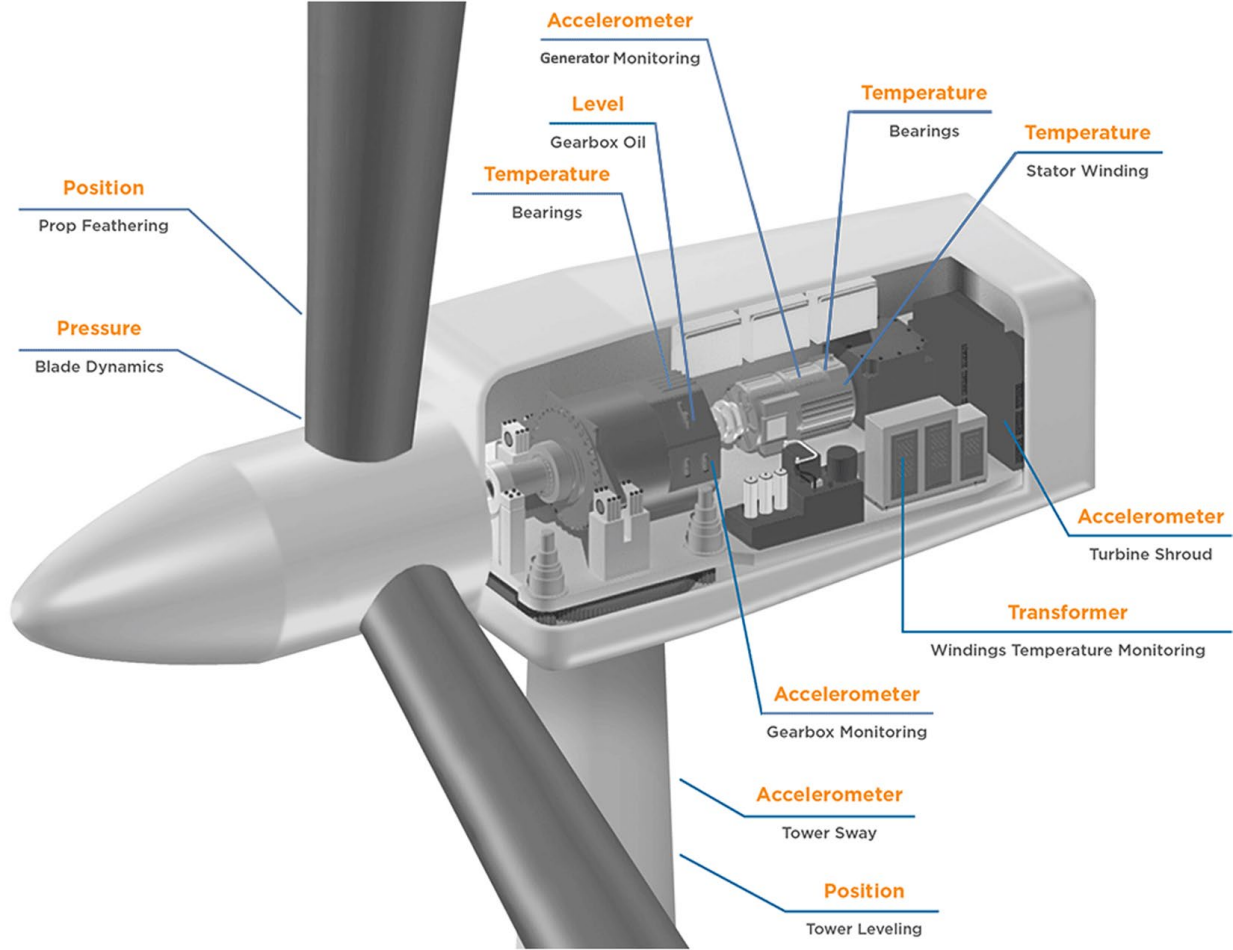
Wind Turbine system and sensors. Adapted from TE connectivity (http://www.te.com/).

The WTs have 78 different sensors (see Fig. 1). Each sensor in the system provides data regarding a specific subsystem and presents summary information in the form of four statistical parameters calculated at 5-minute intervals. Variables from the same sensor can therefore be identified by their common name, with the only distinction being the specific term added: avg (mean), std (standard deviation), min (minimum), or max (maximum). In total, there are 78 subsystems, giving a total of 78 multiplied by 4, which equals a collection of unique variables. For a complete list of names and corresponding variables, see Supplementary Table 2.

The database also provides a comprehensive collection of alarm information. A total of 369 alarms have been identified, each of which is assigned a unique (integer) code and associated with a specific system and its corresponding subsystem, accompanied by a short descriptor. This allows the database user to easily retrieve the set of alarm codes specifically linked to a particular system of interest. This information is available in Supplementary Table 3.

### Gearbox

In the WT subsystems as a whole, the gearbox is a system to be monitored because a broken or damaged gearbox is a serious and costly breakdown with prolonged downtime. The gearbox is a device that increases the speed of the slow but powerful rotation of the rotor to an optimal level for the generator. This allows the generator to convert the maximum mechanical energy of the wind into electricity. During this energy transformation, the gears of the gearbox are stressed due to the difference between the input torque and the opposite torque of the generator at the output. As a result, some parts of the gearbox experience fatigue and an increase in temperature, which hinders the effectiveness of lubrication. Detecting gearbox failures, especially in the early stages, can be difficult. In many cases, a failure in the gearbox involves the replacement of the component with a new one. This failure can be of slow onset (degradation) and, in that case, can be predicted before total failure (breakage).

In a WT gearbox, the transmission is organised in three main parts: the planetary stage, the intermediate stage, and the high-speed stage. Each stage consists of specific components, basically gear parts with a different number of teeth, which allow the speed of the rotor to be adjusted to the generator Fig. 1.

**Fig. 2:**
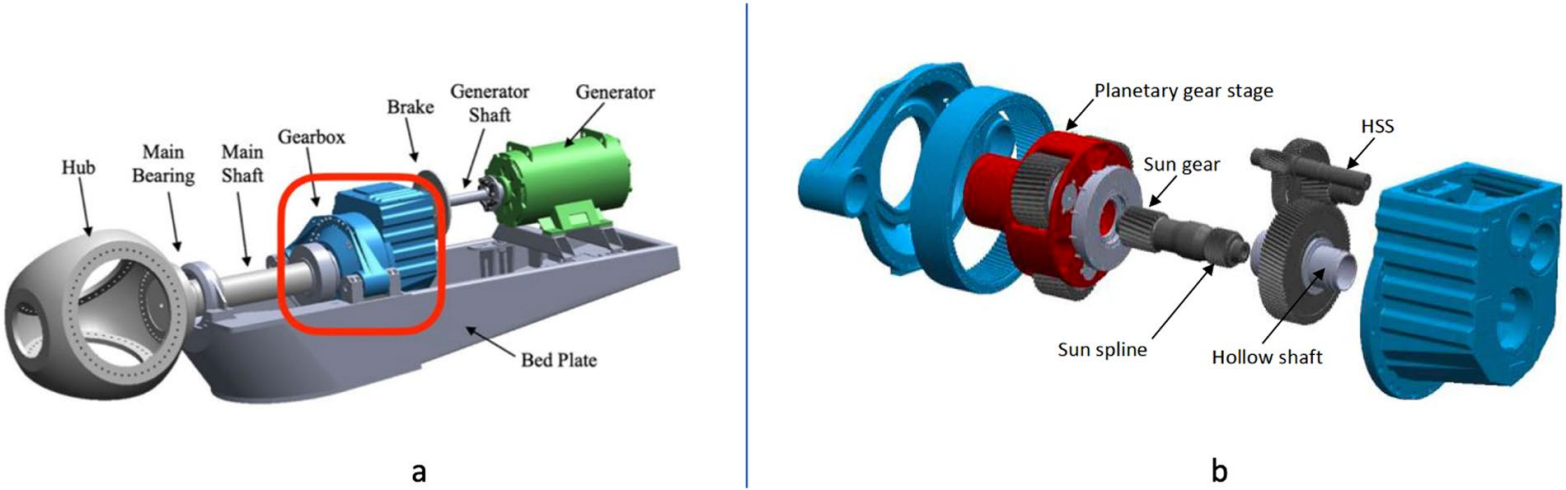
(**a**) Drivetrain configuration. (**b**) Gearbox configuration.

Figure 2a shows a two-speed, stall-controlled, three-bladed upwind turbine drivetrain, in which the gearbox subsystem is shown within a red box. The main components of the drivetrain are the hub, main bearing, main shaft, gearbox, brake, generator shaft and generator. In Fig. 2b, the gearbox is depicted in more detail, showing various types of bearings used depending on the load conditions and gearbox life requirements. In this example, the planetary gearbox is supported by two cylindrical roller bearings (fcCRB), and each planetary gear is supported by two identical cylindrical roller bearings (CRB). Each gearbox parallel shaft is supported by a CRB on the upwind side of the assembly and by two tapered roller bearings (TRB) mounted back-to-back on the leeward side.

### Regulations and standards

The International Electrotechnical Commission (IEC) standards 61400-25 series provide comprehensive guidelines for monitoring and control systems in wind power plants. These standards are particularly relevant in the context of failure detection in wind turbines, especially when considering the implementation of IEC61400 as a SCADA naming standard in wind farms. A detailed examination of each standard’s significance is essential for framing research in this area. The most important ones are:

- IEC 61400-25-2:2015 - Information Models: This standard specifies information models related to wind power plants. The information models typically include the four attributes detailed in Table 1.

**Table 1:** Attributes of the information models.

Data Attributes	Represent specific pieces of information, like wind speed, turbine rotational speed, power output, and temperature. Each attribute is defined with a specific data type and range.
Object Models	Collections of data attributes and methods that represent a specific component of the wind turbine, such as a blade, gearbox, or generator. Object models define how data is organized and related within the system.
Hierarchical Structure	This structure facilitates the organization and retrieval of data, ensuring that the relationships between different components of the wind turbine are logically represented.
Standardized Naming Conventions	Specifies naming conventions for different data elements. This uniformity is crucial for interoperability and easy integration with various SCADA systems.
- IEC 61400-25-3:2015 - Information Exchange Models: Focusing on methods and models for information exchange within wind power plants, this standard outlines essential protocols and communication patterns for efficient data transfer. The most important aspects are listed in Table 2.

**Table 2:** Protocols and communication patterns.

Communication Protocols	Protocols and methods for data exchange, ensuring compatibility and efficiency in communication between different systems and devices.
Data Exchange Patterns	The standard outlines various patterns of data exchange such as request-response, publish-subscribe, and report by exception. These patterns define how data is transmitted, received, and processed.
Security and Reliability	Aspects of secure and reliable data transmission are covered. This includes encryption, authentication, and error-checking mechanisms to ensure that data exchange is secure and error-free.
Interoperability Guidelines	Provides guidelines for ensuring that different systems and devices can work together seamlessly. This is especially important in environments where components from different manufacturers need to communicate with each other.
Real-time Data Exchange	It emphasizes the capability for real-time data exchange, which is critical in operational monitoring and control, as well as in failure detection and response scenarios.
- IEC 61400-25-4:2008 - Mapping to Communication Profile: This standard addresses the mapping of information and exchange models to specific communication profiles.
- IEC 61400-25-5:2006 - Conformance Testing: Providing guidelines for testing the conformance of wind power plants to the IEC 61400-25 series, this standard is essential for validating that the monitoring and control systems adhere to international standards.
- IEC 61400-25-6:2010 - Logical Node Classes and Data Classes for Condition Monitoring: This part of the series specifies logical node classes and data classes for condition monitoring of wind power plants.

In the context of the IEC 61400-25-2:2015 standard, labels or naming conventions for various parameters are standardized to ensure consistency and interoperability. For parameters such as Gearbox Temperature, Main Bearing Temperature or Active Power, the labels would typically adhere to a structured format that includes several components to accurately describe the data point. Here are some examples of how these labels might be structured:

- Gearbox Temperature:
  Label: *WTurbine1.GBX.Temp*
  *WTurbine1* is the wind turbine identifier.
  *GBX* is an abbreviation for Gearbox.
  *Temp* indicates temperature measurement.
- Main Bearing Temperature:
  Label: *WTurbine1.MBear.Temp*
  *WTurbine1* is the wind turbine identifier.
  *MBear* is an abbreviation for Main Bearing.
  *Temp* indicates temperature measurement.
- Active Power:

Label: *WTurbine1.Gen.PwrAct*

*WTurbine1* is the wind turbine identifier.

*Gen* is an abbreviation for Generator.

*PwrAct* indicates Active Power measurement.

These labels are illustrative and follow a logical format, but the exact naming convention may vary depending on the specific implementation and configuration of the SCADA system in use. The key is to maintain a consistent and descriptive naming scheme that aligns with the guidelines of the IEC 61400-25-2:2015 standard, facilitating clear identification and management of data points across the wind power plant’s monitoring and control systems.

## Data Records

The data is available in the repository located at the Figshare repository^25^, but also made available on GitHub at https://github.com/alecuba16/fuhrlander. It offers a valuable opportunity to access the raw, unprocessed data generated by the wind farm’s SCADA system. Unlike many publicly available datasets, which often offer limited or filtered information, our dataset contains the complete, unaltered data directly downloaded from the server. We have deliberately chosen not to pre-process the dataset so that users can explore and analyse the data with methods of their choice. The dataset, stored within the ‘dataset’ folder, is in JSON format. It encompasses a comprehensive collection of data obtained from five wind turbines, specifically turbines 80, 81, 82, 83, and 84, spanning a duration of three years from 2012 to 2014. However, there is a data gap due to a temporary failure of the SCADA system. This is something that may occur in real applications, making it more difficult to work in these environments. The recorded data has a frequency of 5 minutes, encompassing four indicators for each of the 78 sensors, ultimately resulting in a total of 312 variables. For every 5-minute interval, the dataset includes minimum, maximum, mean, and standard deviation values for each sensor, providing a comprehensive overview of their respective measurements.

Table 3 presents a partial extract of the variables, showing the identifier (ID), the time (TIME) and the weather systems (MET). The complete list of variables is given in Supplementary Table 2.

**Table 3:** Extract from the list of variables, divided into systems and variable groups, for the ID, TIME and WMET systems.

System	Variable Group	Stat Type	Signal Name
ID	id	single	turbine_id
TIME	time	single	date_time
WMET	wmet_MetAlt1_Hum	min	wmet_min_MetAlt1_Hum
		avg	wmet_avg_MetAlt1_Hum
		sdv	wmet_sdv_MetAlt1_Hum
		max	wmet_max_MetAlt1_Hum
	wmet_DewPTmp	min	wmet_min_DewPTmp
		avg	wmet_avg_DewPTmp
		sdv	wmet_sdv_DewPTmp
		max	wmet_max_DewPTmp
	wmet_MetAlt1_Press	min	wmet_min_MetAlt1_Press
		avg	wmet_avg_MetAlt1_Press
		sdv	wmet_sdv_MetAlt1_Press
		max	wmet_max_MetAlt1_Press

Additionally, the dataset incorporates valuable information regarding warning and alarm events. These events are accompanied by details specifying the affected system and subsystem, along with concise descriptions elucidating the nature of the event. This supplementary information enhances the dataset’s value by offering insights into potential system anomalies and critical occurrences.

Table 4 contains the 15 initial alarms recorded in the dataset. Each row represents an individual alarm, with an identifier (ID) represented by an integer value. In addition, the table indicates the system (SYS) and subsystem (SUBS) associated with the occurrence of each alarm. Lastly, a concise description (DESC), provided by the system, is included to give more context and information about the alarm event.

**Table 4:** List of the first 15 alarms provided by the manufacturer.

ID	SYS	SUBS	DESC
0	Turbine	Control Cabinet	System OK
5	Turbine	Control Cabinet	Vibration
7	Turbine	Control Cabinet	Turbine is serviced
9	Turbine	Control Cabinet	Remote stop
13	Turbine	Control Cabinet	Manual stop
16	Turbine	Control Cabinet	Emer.stop cont.panel
23	Turbine	Control Cabinet	Repeating error
30	Nacelle	Control Cabinet	Nacelle temp.
31	Nacelle	Control Cabinet	Nacelle temp. stop
41	Turbine	Control Cabinet	UPS battery low
45	Turbine	Power Cabinet	Main ctrl. Supply
55	Turbine	Control Cabinet	Main ctrl.man.reboot
66	Turbine	Control Cabinet	Fire alarm
93	Turbine	Control Cabinet	Service hatch
100	Turbine	Control Cabinet	Repeated grid error

Each alarm is identified by a numeric ID. Note that ID numbers are integers, ordered from lowest to highest, but not consecutive.

## Technical Validation

The dataset originates from a historical dump of the Smartive company’s RDS, a relational database system used to store information from various monitored wind plants. The RDS tables were updated through a push mechanism using an OPC standard driver connected to the Wind Farm. Specifically, the wind farms were equipped with the Mita-teknik SCADA platform^26^, which notified the driver whenever a variable aggregation of 5 minutes was completed with its value. The Smartive-developed driver then stored the value in the RDS historical table. This data path represents the sole source of truth, as there is no alternative means to access the raw wind turbine data. The engineer responsible for the driver implemented a range check based on the SCADA variable information endpoint provided by the mita-teknik hub before storing the raw data. To facilitate the use of this dataset, we have made it publicly available in a self-contained JSON format, eliminating the need for third-party software or specific drivers like parquet or SQL. To validate the dataset’s accuracy, we performed a comparison with the original RDS by sampling several rows from different turbines and time intervals.

## Usage Notes

We downloaded the package *fuhrlander-master.zip* from the repository^25^ or the GitHub located at https://github.com/alecuba16/fuhrlander. After downloading the package, we proceeded to extract its contents directly into the designated working directory. The extracted content included the following files: *LICENSE*, which contains the licensing information, and *README.md*, which provides instructions and essential information about the package, the python program *export_variable_info_from_json.py* and the directories *dataset, matlab* and *r*.

The program called *export_variable_info_from_json.py* is used to extract alarm information from the dataset. Within the *dataset* directory, you will find five zipped files, along with a single file in.json format. This particular.json file contains comprehensive information about the wind farm, including details related to alarms and wind turbines. Each of the zipped files contains individualised data for a specific wind turbine.

To facilitate the use of the information in the database, we have provided additional resources in the form of functions and examples. These resources are located in the directories *matlab* and *r*, respectively. The *matlab* directory contains functions and examples specifically designed to support the use of database information in the MATLAB environment, while the *r* directory provides corresponding resources for use in the R programming language.

To exploit the full potential of the database and the accompanying functions described in this section, we have prepared four illustrative examples. Although the examples have been implemented in MATLAB, they can easily be adapted for use in Python or R programming environments.

The first example demonstrates the necessary dependencies and paths that must be built into the development environment to efficiently access the supplied data and functions. This example assumes that the directory structure provided in the repository remains intact after unpacking. By following the prescribed directory structure, developers can easily access the data and functions needed to facilitate their work.

As a quick start to help users use the dataset, we have implemented outlier detection methods in the repository, as detailed in Section 1 of the Supplementary Material. Users can choose between two options: (i) to remove values outside the range mean ± 3 standard deviations, or (ii) to remove values outside the range median ± 2 absolute deviations from the median. This process is applied to each block of data and the remaining data are used to calculate the mean or the median, depending on the user’s choice. However, users can explore alternative strategies by downloading the data without applying any pre-processing.

The dataset includes all subsystem variables, sorted according to the time events they capture. In addition, warnings and alarms that occurred during the period are provided separately. Users have the possibility to download only the variable data or both the variable data and the associated warnings and alarms, as described in Section 1 of the Supplementary Material.

This dataset has already been used in a number of previous works, demonstrating its value in improving maintenance strategies. For more information on the content and potential applications of the dataset, we encourage users to consult the relevant publications^22–24,27^). These resources will provide valuable context and guidance on how to make the most of the dataset.

In the second example, we illustrate the process of reading the file named *wind_plant_data.json* and storing the wind farm information in a MATLAB structure. In this particular example, we will refer to the structure as ‘WF’. Next, we illustrate how to retrieve the alarm identifiers associated specifically with the ‘Gearbox’ subsystem. By accessing the organised data within the WF structure, a vector of numeric alarm identifiers related to the specified subsystem can be obtained. This approach can be similarly applied to acquire information on alarm identifiers linked to any wind turbine system or subsystem.

The third example shows how to obtain the raw data of the WT80. This data is compressed in the file *turbine_80.json.bz2* and the function *get_turbine_data* decompresses it. The SCADA system of these machines provides the 4 statistical measurements in intervals of 5 minutes, so the Data Rate must be 300 s as it is required in seconds. The data of the desired WT is obtained by simply changing the identifier of each WT (80, 81, 82, 83, 84). The result is a table named WT80 in this example 3.

**Fig. 3:**
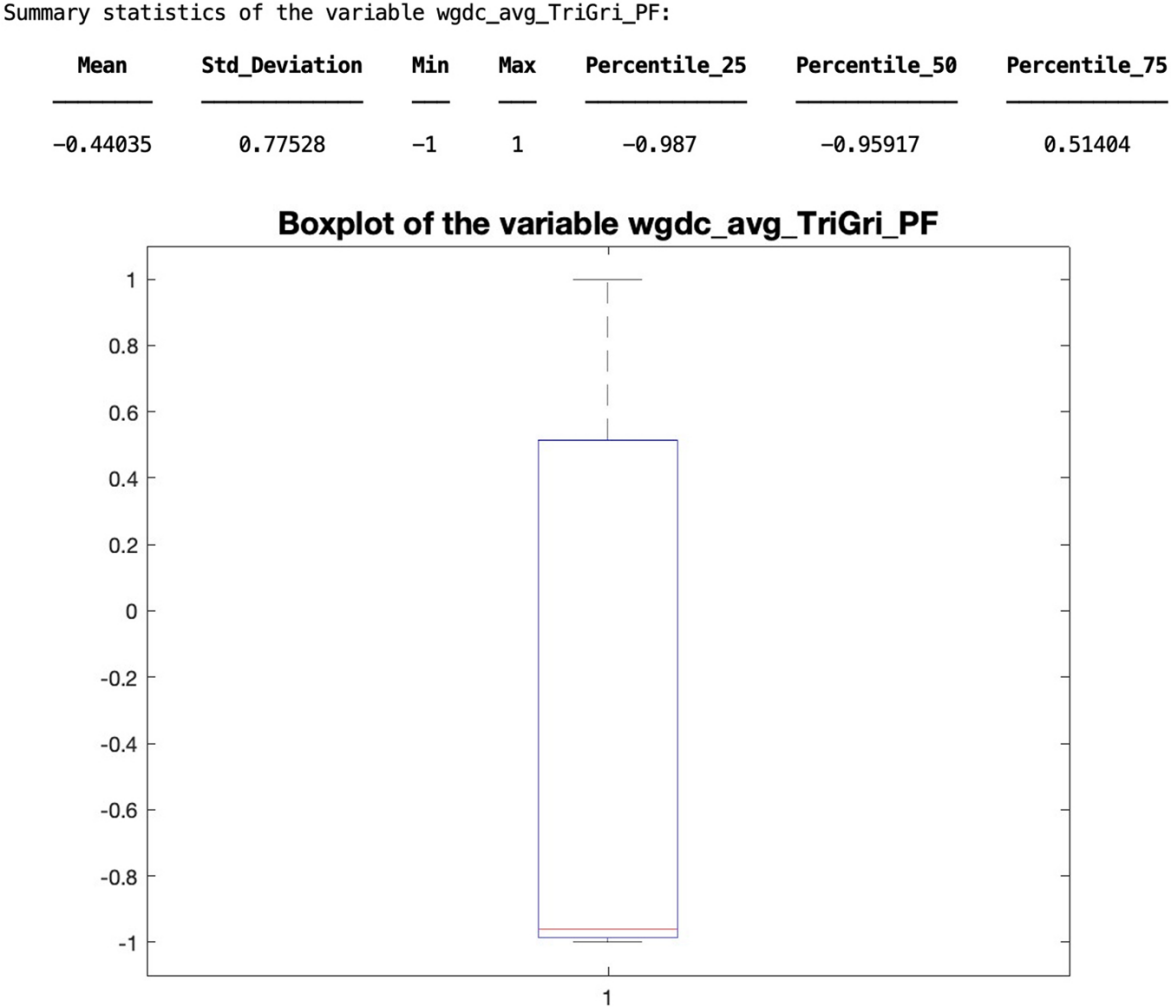
At the top, the table summarises the statistics of the selected variable. At the bottom, the representation of this summary using a boxplot.

Because of the importance of the statistical distribution of variables, the following example explains how to obtain a summary of the statistics of any selected variable. A Matlab function has been prepared for this purpose:

The summary statistics for the variable ‘wgdc_avg_TriGri_PhV_phsA’ of the WT80 can be obtained by the following instruction:

The result generated by the function is shown below (see Fig. 3).

Finally, the last example shows the process of synchronising data and alarm information. From the previous example, we will establish a connection between the selected data of the subsystem ‘Gearbox’ and the data corresponding to the WT80 by means of the function *get_turbine_data*.

There are two different methods to link alarms and data. In this particular case, the output of the function will be in table format. The initial 312 columns of the table represent the 312 signals derived from the SCADA system of WT80. These alarm-related columns are coded in binary format, with a value of 0 indicating no alarm activation and a value of 1 indicating alarm activation.

It is important to note that the function *get_turbine_data* has additional functionalities beyond the linking of data and alarms. In the context of this example 5, in addition to establishing the link between data and alarms, the information is integrated in time intervals of one hour (3600 seconds). Furthermore, the ‘filtered_3sdv_mean’ option is used to filter outliers present in all variables using the 3*σ*-rule.

Options and possibilities of the *get_turbine_data* function can be found in the Supplementary Material.

### Illustrative example

In this section, we will present the results of a simple normality model applied to the WT84 turbine. It is known that this turbine experienced a major failure and remained out of service for a long period of time. Our aim is to demonstrate how a normality model, built using Extreme Learning Machines (ELM) as discussed in^23^, can effectively anticipate and detect such incidents at an advanced stage.

In this example, we will illustrate how a part of the database can be used to develop a model that estimates a target variable within the gearbox subsystem based on a set of related variables. It is important to note that the gearbox has the longest downtime in the event of a failure. Despite the maturity and reliability of the manufacturing technology, this subsystem is prone to breakdowns and failures within a 5-year operating period due to the demanding operating conditions. Apart from the replacement costs, gearbox failure causes system downtime, which lengthens repair times, as it is one of the slowest systems to repair. The cost of gearbox replacement can be up to 14.5% of the maintenance cost of the wind turbine^28^. Consequently, gearbox failure prediction becomes a top priority, which is why it has been selected for this example.

Normality models aim to identify the point at which model predictions deviate from actual values, serving as a potential indicator of failure. In a previous study on ELMs applied to WT condition monitoring, the use of ELMs was explored^23,27^. Accordingly, we adopted the same network structure used in that work. The set of selected variables and the target variable for building the normality model can be found in Table 5. It should be noted that the target variable and the variables used remain consistent with those used in^23^, although their application may differ. This section will show the same target variable as in the previous study.

**Table 5:** List of variables selected to feed the ELM and the estimated target to monitor the gearbox subsystem.

Variable Name	Description
wgdc_avg_TriGri_PwrAt	Transformer, grid side, active power
wgdc_avg_TriGri_PF	Transformer, grid side, power factor
wtrm_avg_TrmTmp_GbxOil	Temperature of the gearbox oil, in degrees Celsius.
wgen_avg_RtrSpd_WP2035	Speed of the rotor main shaft before gearbox in RPM
wnac_avg_WSpd1	Wind speed in m/s measured by the anemometer at the WT’s nacelle
wtrm_avg_TrmTmp_GbxOil	Temperature of the gearbox oil
wnac_avg_ExlTmp	External temperature
wtrm_avg_TrmTmp_GbxBrg151	Speed of the rotor main shaft before gearbox in the point 151
wtrm_avg_TrmTmp_GbxOil	Temperature of the gearbox oil
**Target Name**	**Description**
wtrm_avg_TrmTmp_GbxBrg152	Temperature of the gearbox bearing 152, at the high speed shaft (output)

Since the aim is to demonstrate the usefulness of the released database rather than to carry out an exhaustive study of it, we will focus on presenting the results for the WT84 turbine only. This particular turbine suffered a major failure and was out of service for a long period of time. Therefore, we will use the WT84 turbine to train a model and test it when it is working properly, as well as before the failure, in order to illustrate how simple models can be used to monitor wind farms and often predict critical failures. This approach allows us to see how SCADA data can be used to detect the early stages of system deterioration.

Specifically, the model is trained using the first 25% of the initial WT84 data. The remaining data (75%) is used for various tests. The model consists of a feed-forward network with *H* = 50 hidden nodes and a sigmoid activation function, following the architecture presented in^23^. The model is not optimised and no formal feature selection is performed. The choice of variables is made intuitively based on knowledge generated in previous studies, as the problem was studied in^23^. The optimal network size (number of hidden nodes) is also not explored. The initial calculation of the ELM output weights is the solution presented, as different realisations yield similar results.

Since the ELM technique is robust to moderate outliers, no outlier filtering is performed in this example. The only processing applied to the raw data is a z-score normalisation of the variables and the target. The obtained normalisation constants (mean and variance) will be used to normalise and denormalise the test data.

Therefore, it is a simpler example compared to the one included in the repository (*example_elm.m*), and it can be performed using the methods of the implemented ELM class *elm_classifier.m*, also included in the repository, which facilitates the development and testing of the model.

**Fig. 4:**
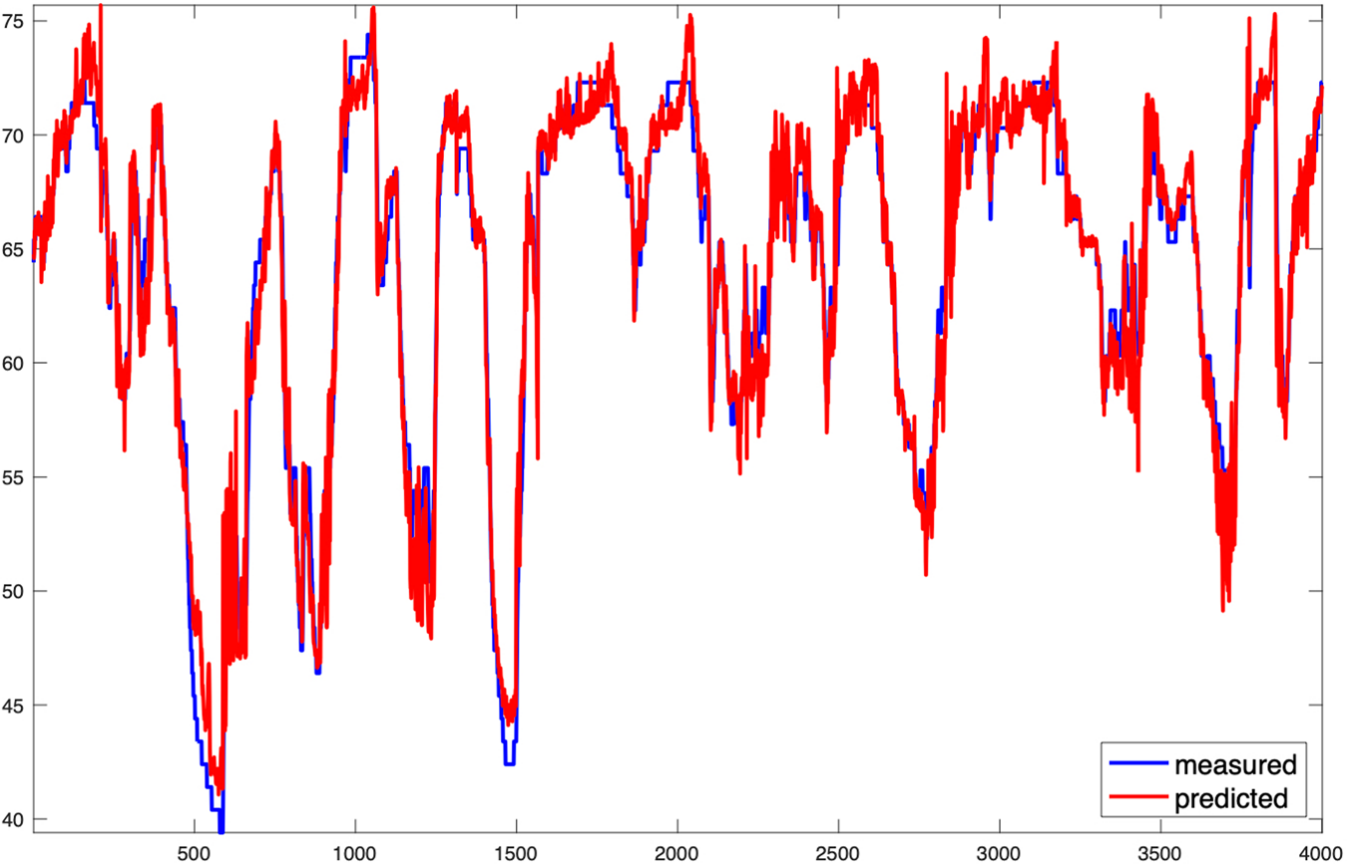
Time interval of the test phase in which the model follows the signal. The horizontal axis represents the number of samples and the vertical axis the temperature in degrees Celsius.

**Fig. 5:**
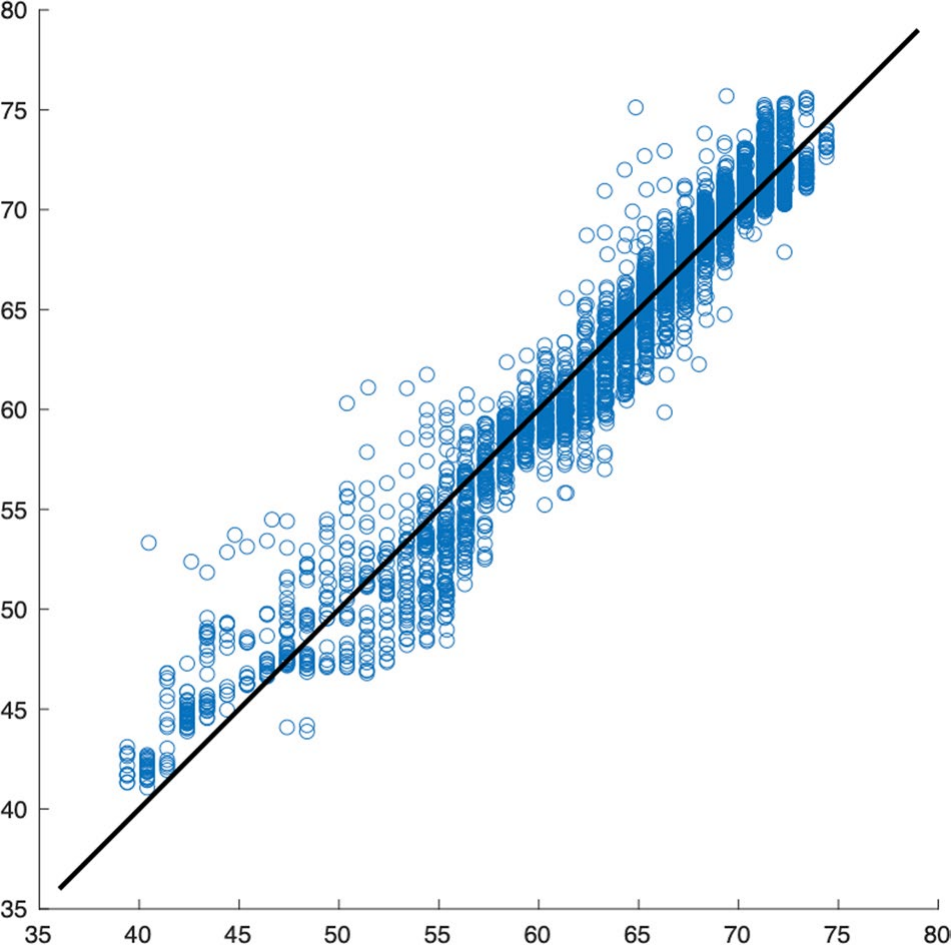
Scatter plot of the above results, where the model follows the signal. The horizontal axis represents the measured signal and the vertical axis the estimated signal. The identity function is shown in black.

**Fig. 6:**
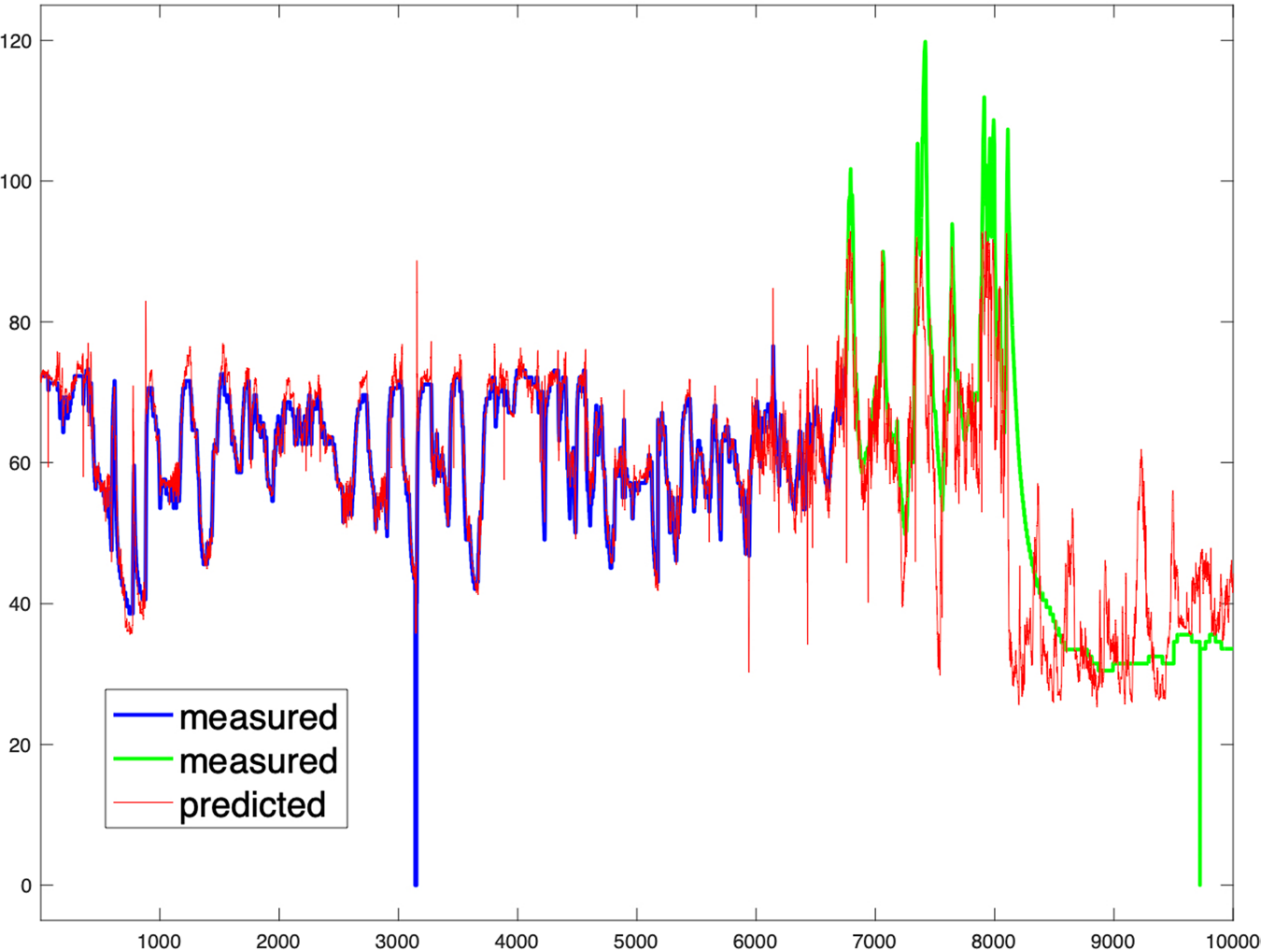
Representation of the estimated and measured target within the time window that captures the onset of gear system malfunction and system failure. Note that the mismatch between the model and the system starts to be observed before sample 7000 (measured values depicted in blue color), while the failure occurs around sample 8000, after which the WT84 stops working and the target measurement tends toward room temperature values (measured values depicted in green color). Taking into account that each sample is spaced 5 minutes apart, the detection of anomalies 1000 samples (5000 minutes) before the failure indicates that the first signs could have been observed approximately 17 days before the event.

The main difference from the *example_elm.m* model is our specific focus on WT84. We develop a dedicated model exclusively for this turbine, with a single iteration (unlike the multiple iterations used in the example to calculate output weights and identify the best solution). Additionally, we limit the training data to only the first 25% of the available data for WT84, obtained through Example 3, without incorporating any failure data. Since no optimization has been performed, we do not present accuracy measures or make other comparisons. In this context, our presentation includes a time segment during which WT84 operates correctly. Figure 4 displays this time segment, showcasing the model estimate alongside the actual measurements. Additionally, Fig. 5 presents the corresponding regression model for this segment. Furthermore, Fig. 6 illustrates the representation of the estimate and the target over a time interval spanning both before and after the gearbox failure. The failure caused a breakdown, leading to an extended period of WT84 being out of service. In the figure, the first 7000 samples, denoted in blue, represent the actual measured values. The remaining samples, depicted in green, correspond to the data points when WT84 was not functioning correctly.

**Fig. 7:**
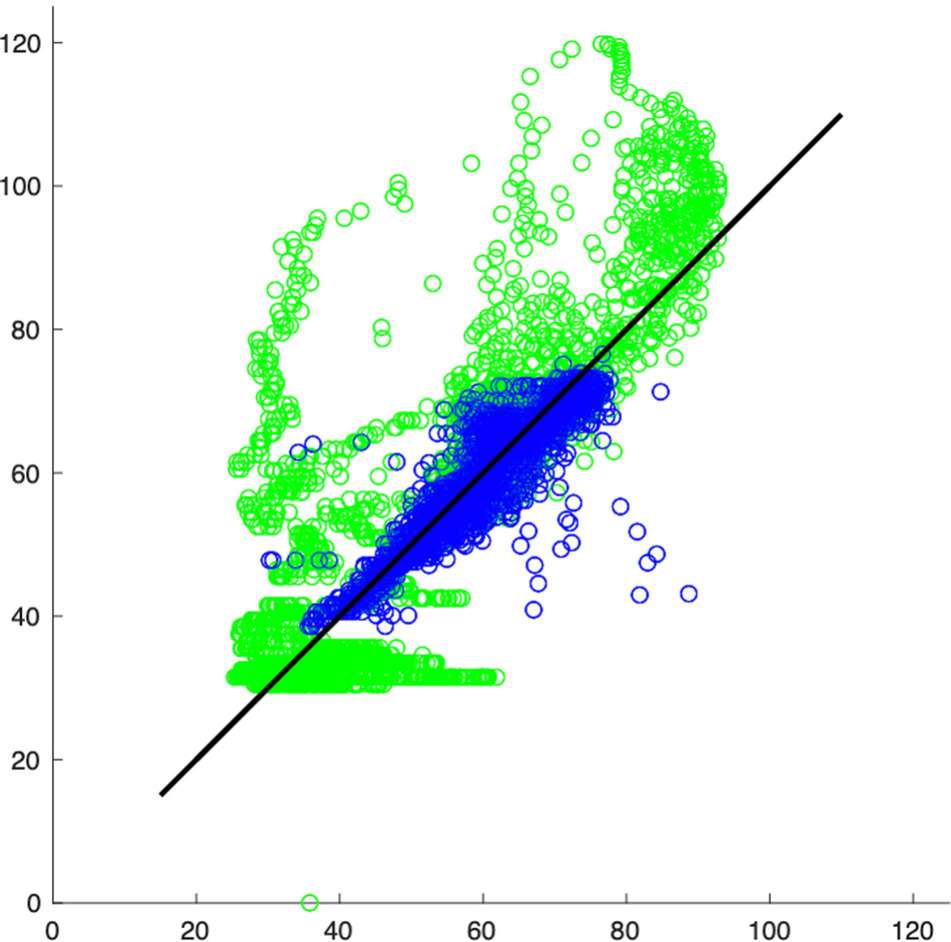
Scatter plot of the above results, where the model starts to diverge of the signal. The horizontal axis represents the measured signal and the vertical axis the estimated signal. The identity function is shown in black. Blue dots correspond to the first 7000 points of the measured variable (shown in blue in Fig. 5), in which the WT84 works properly. Green points correspond to the last 3000 points (shown in green in Fig. 5) in which the WT84 had a failure. It can clearly be seen that these (green) measured values are far away from the values predicted by the ELM model.

For a clearer visualization of the disparity between the real measured target and the one predicted by the EML model, refer to Fig. 7. In this figure, the blue points represent the first 7000 samples, where the model produces an accurate estimate of the target. On the other hand, the green points correspond to the remaining samples, where the model noticeably deviates from the target due to the malfunction of WT84 during that period.

### Supplementary information

Supplementary information

## Data Availability

The turbine dataset was generated by aggregating the SCADA data obtained from the entire wind farm. It consists of five wind turbines, all of them of the same model and manufacturer: Fuhrländer FL2500 2.5 MW. To facilitate the manipulation and pre-processing of the data, we have developed functions in the programming languages R and MATLAB to serve as an interface. These functions efficiently transform the raw data into a structured table format. In this format, each variable corresponds to a column, while each entry represents a five-minute interval of data recorded in the rows. The database and the code are freely available at^25^ and at the GitHub page https://github.com/alecuba16/fuhrlander.

## References

[1] Eurostat. Energy balance sheets 2011–2012, 10.2785/52802 (2014).

[2] Milborrow D (2006). Operation and maintenance costs compared and revealed. Wind Stats.

[3] Lei, X., Sandborn, P., Bakhshi, R., Kashani-Pour, A. & Goudarzi, N. Phm based predictive maintenance optimization for offshore wind farms. In *2015 IEEE conference on prognostics and health management (PHM)*, 1–8, 10.1109/icphm.2015.7245027 (IEEE, 2015).

[4] Hahn, B., Durstewitz, M. & Rohrig, K. Reliability of wind turbines. In *Wind Energy: Proceedings of the Euromech Colloquium*, 329–332, 10.1007/978-3-540-33866-6_62 (Springer, 2007).

[5] Ziegler L, Gonzalez E, Rubert T, Smolka U, Melero JJ (2018). Lifetime extension of onshore wind turbines: A review covering germany, spain, denmark, and the uk. Renewable and Sustainable Energy Reviews.

[6] Ding F, Tian Z, Zhao F, Xu H (2018). An integrated approach for wind turbine gearbox fatigue life prediction considering instantaneously varying load conditions. Renewable energy.

[7] Corley, B., Carroll, J. & Mcdonald, A. Fault detection of wind turbine gearbox using thermal network modelling and scada data. In *Journal of Physics: Conference Series*, vol. 1618, 022042, 10.1088/1742-6596/1618/2/022042 (IOP Publishing, 2020).

[8] Corley B, Koukoura S, Carroll J, McDonald A (2021). Combination of thermal modelling and machine learning approaches for fault detection in wind turbine gearboxes. Energies.

[9] Soua S, Van Lieshout P, Perera A, Gan T-H, Bridge B (2013). Determination of the combined vibrational and acoustic emission signature of a wind turbine gearbox and generator shaft in service as a pre-requisite for effective condition monitoring. Renewable Energy.

[10] Siegel D, Zhao W, Lapira E, AbuAli M, Lee J (2014). A comparative study on vibration-based condition monitoring algorithms for wind turbine drive trains. Wind energy.

[11] Artigao E (2018). Current signature and vibration analyses to diagnose an in-service wind turbine drive train. Energies.

[12] Zheng C (2020). Science Data Bank.

[13] Zhang T, Tian B, Sengupta D, Zhang L, Si Y (2021). Global offshore wind turbine dataset. Scientific Data.

[14] Effenberger N, Ludwig N (2022). A collection and categorization of open-source wind and wind power datasets. Wind Energy.

[15] Lu B, Durocher DB, Stemper P (2009). Predictive maintenance techniques. IEEE Industry Applications Magazine.

[16] Márquez FPG, Tobias AM, Pérez JMP, Papaelias M (2012). Condition monitoring of wind turbines: Techniques and methods. Renewable energy.

[17] Tautz-Weinert J, Watson SJ (2017). Using scada data for wind turbine condition monitoring–a review. IET Renewable Power Generation.

[18] Stetco A (2019). Machine learning methods for wind turbine condition monitoring: A review. Renewable energy.

[19] Yang W, Court R, Jiang J (2013). Wind turbine condition monitoring by the approach of scada data analysis. Renewable energy.

[20] Zaher A, McArthur S, Infield D, Patel Y (2009). Online wind turbine fault detection through automated scada data analysis. Wind Energy: An International Journal for Progress and Applications in Wind Power Conversion Technology.

[21] Gonzalez E, Stephen B, Infield D, Melero JJ (2019). Using high-frequency scada data for wind turbine performance monitoring: A sensitivity study. Renewable energy.

[22] Marti-Puig P, Blanco-M A, Cárdenas JJ, Cusidó J, Solé-Casals J (2019). Feature selection algorithms for wind turbine failure prediction. Energies.

[23] Marti-Puig P, Blanco-M A, Serra-Serra M, Solé-Casals J (2021). Wind turbine prognosis models based on scada data and extreme learning machines. Applied Sciences.

[24] Marti-Puig P (2022). Detection of wind turbine failures through cross-information between neighbouring turbines. Applied Sciences.

[25] Martínez AB, Solé-Casals J, Marti-Puig P (2024). Figshare.

[26] *Wind Park Control Concept Manual*. https://www.mita-teknik.com/media/1141/binder1.pdf.

[27] Marti-Puig P, Bennásar-Sevillá A, Blanco-M A, Solé-Casals J (2021). Exploring the effect of temporal aggregation on scada data for wind turbine prognosis using a normality model. Applied Sciences.

[28] Pérez JMP, Márquez FPG, Tobias A, Papaelias M (2013). Wind turbine reliability analysis. Renewable and Sustainable Energy Reviews.

