# Supplementary material for: Wind turbine database for intelligent operation and maintenance strategies
Source: Sci Data. 2024 Feb 29;11:255. doi: 10.1038/s41597-024-03067-9 (PMC10904773; doi:10.1038/s41597-024-03067-9)
Supplement: Supplementary file 1 — Supplementary information [file 41597_2024_3067_MOESM1_ESM.pdf]

Supplementary material

Contents

1 Systems and subsystems 1

2 Exhaustive signals list provided by the SCADA system 1

3 Description of the alarms 7

4 Options of the function *get_turbine_data* 14

1 Systems and subsystems

Table 1 contains the list of the systems and subsystems of the wind turbines. Note that some systems do not have subsystems while others have several. This table is derived directly from the SCADA database.

SYS	SUBS
Converter	Converter
Generator	Generator
Nacelle	Control Cabinet
Rotor blade	Control Cabinet<br>Hub<br>Pitch<br>Rotor
Tower	Control Cabinet<br>Tower
Transformer	Transformer
Transmission	Brake<br>Gearbox<br>Hydraulic System<br>Main Bearing
Turbine	Control Cabinet<br>Power Cabinet<br>Roof
Yaw	Yaw

Table 1. Systems and the subsystems of the wind turbines.

2 Exhaustive signals list provided by the SCADA system

Table 2 contains all the signals recorded by the SCADA system. The first is the system identification, the second column is the variable group, the third column contains the statistics type of the variable, and the last column provides the signal name.

System	Variable Group	Stat Type	Signal Name
ID	id	single	turbine_id
TIME	time	single	date_time
WMET	wmet_MetAlt1_Hum	min	wmet_min_MetAlt1_Hum
		avg	wmet_avg_MetAlt1_Hum
		sdv	wmet_sdv_MetAlt1_Hum
		max	wmet_max_MetAlt1_Hum
	wmet_DewPTmp	min	wmet_min_DewPTmp
		avg	wmet_avg_DewPTmp
		sdv	wmet_sdv_DewPTmp
		max	wmet_max_DewPTmp
	wmet_MetAlt1_Press	min	wmet_min_MetAlt1_Press
		avg	wmet_avg_MetAlt1_Press

		sdv	wmet_sdv_MetAlt1_Press
		max	wmet_max_MetAlt1_Press
WGDC	wgdc_TriGri_PF	min	wgdc_min_TriGri_PF
		avg	wgdc_avg_TriGri_PF
		sdv	wgdc_sdv_TriGri_PF
		max	wgdc_max_TriGri_PF
	wgdc_TriGri_A_phsC	min	wgdc_min_TriGri_A_phsC
		avg	wgdc_avg_TriGri_A_phsC
		sdv	wgdc_sdv_TriGri_A_phsC
		max	wgdc_max_TriGri_A_phsC
	wgdc_LoVTmp	min	wgdc_min_LoVTmp
		avg	wgdc_avg_LoVTmp
		sdv	wgdc_sdv_LoVTmp
		max	wgdc_max_LoVTmp
	wgdc_TriGri_PwrAt	min	wgdc_min_TriGri_PwrAt
		avg	wgdc_avg_TriGri_PwrAt
		sdv	wgdc_sdv_TriGri_PwrAt
		max	wgdc_max_TriGri_PwrAt
	wgdc_GdcTmp_TrfGn	min	wgdc_min_GdcTmp_TrfGn
		avg	wgdc_avg_GdcTmp_TrfGn
		sdv	wgdc_sdv_GdcTmp_TrfGn
		max	wgdc_max_GdcTmp_TrfGn
	wgdc_TriGri_PhV_phsA	min	wgdc_min_TriGri_PhV_phsA
		avg	wgdc_avg_TriGri_PhV_phsA
		sdv	wgdc_sdv_TriGri_PhV_phsA
		max	wgdc_max_TriGri_PhV_phsA
	wgdc_TriGri_PwrReact	min	wgdc_min_TriGri_PwrReact
		avg	wgdc_avg_TriGri_PwrReact
		sdv	wgdc_sdv_TriGri_PwrReact
		max	wgdc_max_TriGri_PwrReact
	wgdc_TriGri_PhV_phsB	min	wgdc_min_TriGri_PhV_phsB
		avg	wgdc_avg_TriGri_PhV_phsB
		sdv	wgdc_sdv_TriGri_PhV_phsB
		max	wgdc_max_TriGri_PhV_phsB
	wgdc_TriGri_Hz	min	wgdc_min_TriGri_Hz
		avg	wgdc_avg_TriGri_Hz
		sdv	wgdc_sdv_TriGri_Hz
		max	wgdc_max_TriGri_Hz
	wgdc_TriGri_A_phsB	min	wgdc_min_TriGri_A_phsB
		avg	wgdc_avg_TriGri_A_phsB
		sdv	wgdc_sdv_TriGri_A_phsB
		max	wgdc_max_TriGri_A_phsB
	wgdc_TriGri_A_phsA	min	wgdc_min_TriGri_A_phsA
		avg	wgdc_avg_TriGri_A_phsA
		sdv	wgdc_sdv_TriGri_A_phsA
		max	wgdc_max_TriGri_A_phsA
	wgdc_TriGri_PhV_phsC	min	wgdc_min_TriGri_PhV_phsC
		avg	wgdc_avg_TriGri_PhV_phsC
		sdv	wgdc_sdv_TriGri_PhV_phsC
		max	wgdc_max_TriGri_PhV_phsC
	wgdc_TriGri_PhV	min	wgdc_min_TriGri_PhV
		avg	wgdc_avg_TriGri_PhV
		sdv	wgdc_sdv_TriGri_PhV
		max	wgdc_max_TriGri_PhV
	wgdc_TriGri_A	min	wgdc_min_TriGri_A

		avg	wgdc_avg_TriGri_A
		sdv	wgdc_sdv_TriGri_A
		max	wgdc_max_TriGri_A
WTOW	wtow_PwrPnlTmp	min	wtow_min_PwrPnlTmp
		avg	wtow_avg_PwrPnlTmp
		sdv	wtow_sdv_PwrPnlTmp
		max	wtow_max_PwrPnlTmp
WTRM	wtrm_Gbx_OilPres	min	wtrm_min_Gbx_OilPres
		avg	wtrm_avg_Gbx_OilPres
		sdv	wtrm_sdv_Gbx_OilPres
		max	wtrm_max_Gbx_OilPres
	wtrm_TrmTmp_GnBrgNDE	min	wtrm_min_TrmTmp_GnBrgNDE
		avg	wtrm_avg_TrmTmp_GnBrgNDE
		sdv	wtrm_sdv_TrmTmp_GnBrgNDE
		max	wtrm_max_TrmTmp_GnBrgNDE
	wtrm_TrmTmp_Brg1	min	wtrm_min_TrmTmp_Brg1
		avg	wtrm_avg_TrmTmp_Brg1
		sdv	wtrm_sdv_TrmTmp_Brg1
		max	wtrm_max_TrmTmp_Brg1
	wtrm_TrmTmp_GbxBrg450	min	wtrm_min_TrmTmp_GbxBrg450
		avg	wtrm_avg_TrmTmp_GbxBrg450
		sdv	wtrm_sdv_TrmTmp_GbxBrg450
		max	wtrm_max_TrmTmp_GbxBrg450
	wtrm_TrmTmp_GbxClWtBkw	min	wtrm_min_TrmTmp_GbxClWtBkw
		avg	wtrm_avg_TrmTmp_GbxClWtBkw
		sdv	wtrm_sdv_TrmTmp_GbxClWtBkw
		max	wtrm_max_TrmTmp_GbxClWtBkw
	wtrm_TrmTmp_GbxClWtFrw	min	wtrm_min_TrmTmp_GbxClWtFrw
		avg	wtrm_avg_TrmTmp_GbxClWtFrw
		sdv	wtrm_sdv_TrmTmp_GbxClWtFrw
		max	wtrm_max_TrmTmp_GbxClWtFrw
	wtrm_TrmTmp_GbxBrg451	min	wtrm_min_TrmTmp_GbxBrg451
		avg	wtrm_avg_TrmTmp_GbxBrg451
		sdv	wtrm_sdv_TrmTmp_GbxBrg451
		max	wtrm_max_TrmTmp_GbxBrg451
	wtrm_Brg_OilPresIn	min	wtrm_min_Brg_OilPresIn
		avg	wtrm_avg_Brg_OilPresIn
		sdv	wtrm_sdv_Brg_OilPresIn
		max	wtrm_max_Brg_OilPresIn
	wtrm_TrmTmp_GnBrgDE	min	wtrm_min_TrmTmp_GnBrgDE
		avg	wtrm_avg_TrmTmp_GnBrgDE
		sdv	wtrm_sdv_TrmTmp_GnBrgDE
		max	wtrm_max_TrmTmp_GnBrgDE
	wtrm_TrmTmp_GbxBrg452	min	wtrm_min_TrmTmp_GbxBrg452
		avg	wtrm_avg_TrmTmp_GbxBrg452
		sdv	wtrm_sdv_TrmTmp_GbxBrg452
		max	wtrm_max_TrmTmp_GbxBrg452
	wtrm_TrmTmp_Brg2	min	wtrm_min_TrmTmp_Brg2
		avg	wtrm_avg_TrmTmp_Brg2
		sdv	wtrm_sdv_TrmTmp_Brg2
		max	wtrm_max_TrmTmp_Brg2
	wtrm_TrmTmp_GbxOil	min	wtrm_min_TrmTmp_GbxOil
		avg	wtrm_avg_TrmTmp_GbxOil
		sdv	wtrm_sdv_TrmTmp_GbxOil

		max	wtrm_max_TrmTmp_GbxOil
	wtrm_TrmTmp_GnClWtFrw	min	wtrm_min_TrmTmp_GnClWtFrw
		avg	wtrm_avg_TrmTmp_GnClWtFrw
		sdv	wtrm_sdv_TrmTmp_GnClWtFrw
		max	wtrm_max_TrmTmp_GnClWtFrw
	wtrm_TrmTmp_GbxBrg151	min	wtrm_min_TrmTmp_GbxBrg151
		avg	wtrm_avg_TrmTmp_GbxBrg151
		sdv	wtrm_sdv_TrmTmp_GbxBrg151
		max	wtrm_max_TrmTmp_GbxBrg151
	wtrm_TrmTmp_Gbx	min	wtrm_min_TrmTmp_Gbx
		avg	wtrm_avg_TrmTmp_Gbx
		sdv	wtrm_sdv_TrmTmp_Gbx
		max	wtrm_max_TrmTmp_Gbx
	wtrm_TrmTmp_GbxBrg152	min	wtrm_min_TrmTmp_GbxBrg152
		avg	wtrm_avg_TrmTmp_GbxBrg152
		sdv	wtrm_sdv_TrmTmp_GbxBrg152
		max	wtrm_max_TrmTmp_GbxBrg152
	wtrm_TrmTmp_GnClWtBkw	min	wtrm_min_TrmTmp_GnClWtBkw
		avg	wtrm_avg_TrmTmp_GnClWtBkw
		sdv	wtrm_sdv_TrmTmp_GnClWtBkw
		max	wtrm_max_TrmTmp_GnClWtBkw
	wtrm_Brg_OilPres	min	wtrm_min_Brg_OilPres
		avg	wtrm_avg_Brg_OilPres
		sdv	wtrm_sdv_Brg_OilPres
		max	wtrm_max_Brg_OilPres
WTUR	wtur_PwrRedCau	min	wtur_min_PwrRedCau
		avg	wtur_avg_PwrRedCau
		sdv	wtur_sdv_PwrRedCau
		max	wtur_max_PwrRedCau
	wtur_PwrRedSp	min	wtur_min_PwrRedSp
		avg	wtur_avg_PwrRedSp
		sdv	wtur_sdv_PwrRedSp
		max	wtur_max_PwrRedSp
	wtur_PwrRedNoi	min	wtur_min_PwrRedNoi
		avg	wtur_avg_PwrRedNoi
		sdv	wtur_sdv_PwrRedNoi
		max	wtur_max_PwrRedNoi
	wtur_ExtPwrSpUtil	min	wtur_min_ExtPwrSpUtil
		avg	wtur_avg_ExtPwrSpUtil
		sdv	wtur_sdv_ExtPwrSpUtil
		max	wtur_max_ExtPwrSpUtil
	wtur_ExtPwrReactSp	min	wtur_min_ExtPwrReactSp
		avg	wtur_avg_ExtPwrReactSp
		sdv	wtur_sdv_ExtPwrReactSp
		max	wtur_max_ExtPwrReactSp
WROT	wrot_TmpPwrSply_ValBl2	min	wrot_min_TmpPwrSply_ValBl2
		avg	wrot_avg_TmpPwrSply_ValBl2
		sdv	wrot_sdv_TmpPwrSply_ValBl2
		max	wrot_max_TmpPwrSply_ValBl2
	wrot_TmpHtSinkPco_Bl2	min	wrot_min_TmpHtSinkPco_Bl2
		avg	wrot_avg_TmpHtSinkPco_Bl2
		sdv	wrot_sdv_TmpHtSinkPco_Bl2
		max	wrot_max_TmpHtSinkPco_Bl2
	wrot_TmpCpt_Bl3	min	wrot_min_TmpCpt_Bl3
		avg	wrot_avg_TmpCpt_Bl3

		sdv	wrot_sdv_TmpCpt_Bl3
		max	wrot_max_TmpCpt_Bl3
	wrot_A_ValBl2	min	wrot_min_A_ValBl2
		avg	wrot_avg_A_ValBl2
		sdv	wrot_sdv_A_ValBl2
		max	wrot_max_A_ValBl2
	wrot_TmpHtSinkPco_Bl1	min	wrot_min_TmpHtSinkPco_Bl1
		avg	wrot_avg_TmpHtSinkPco_Bl1
		sdv	wrot_sdv_TmpHtSinkPco_Bl1
		max	wrot_max_TmpHtSinkPco_Bl1
	wrot_TmpCpt_Bl2	min	wrot_min_TmpCpt_Bl2
		avg	wrot_avg_TmpCpt_Bl2
		sdv	wrot_sdv_TmpCpt_Bl2
		max	wrot_max_TmpCpt_Bl2
	wrot_A_ValBl3	min	wrot_min_A_ValBl3
		avg	wrot_avg_A_ValBl3
		sdv	wrot_sdv_A_ValBl3
		max	wrot_max_A_ValBl3
	wrot_TmpPwrSply_ValBl1	min	wrot_min_TmpPwrSply_ValBl1
		avg	wrot_avg_TmpPwrSply_ValBl1
		sdv	wrot_sdv_TmpPwrSply_ValBl1
		max	wrot_max_TmpPwrSply_ValBl1
	wrot_A_ValBl1	min	wrot_min_A_ValBl1
		avg	wrot_avg_A_ValBl1
		sdv	wrot_sdv_A_ValBl1
		max	wrot_max_A_ValBl1
	wrot_V_ValBl2	min	wrot_min_V_ValBl2
		avg	wrot_avg_V_ValBl2
		sdv	wrot_sdv_V_ValBl2
		max	wrot_max_V_ValBl2
	wrot_V_ValBl1	min	wrot_min_V_ValBl1
		avg	wrot_avg_V_ValBl1
		sdv	wrot_sdv_V_ValBl1
		max	wrot_max_V_ValBl1
	wrot_TmpHtSinkPco_Bl3	min	wrot_min_TmpHtSinkPco_Bl3
		avg	wrot_avg_TmpHtSinkPco_Bl3
		sdv	wrot_sdv_TmpHtSinkPco_Bl3
		max	wrot_max_TmpHtSinkPco_Bl3
	wrot_TmpPDU_ValBl2	min	wrot_min_TmpPDU_ValBl2
		avg	wrot_avg_TmpPDU_ValBl2
		sdv	wrot_sdv_TmpPDU_ValBl2
		max	wrot_max_TmpPDU_ValBl2
	wrot_RotSt_Bl1_PDU	min	wrot_min_RotSt_Bl1_PDU
		avg	wrot_avg_RotSt_Bl1_PDU
		sdv	wrot_sdv_RotSt_Bl1_PDU
		max	wrot_max_RotSt_Bl1_PDU
	wrot_TmpCpt_Bl1	min	wrot_min_TmpCpt_Bl1
		avg	wrot_avg_TmpCpt_Bl1
		sdv	wrot_sdv_TmpCpt_Bl1
		max	wrot_max_TmpCpt_Bl1
	wrot_TmpPDU_ValBl3	min	wrot_min_TmpPDU_ValBl3
		avg	wrot_avg_TmpPDU_ValBl3
		sdv	wrot_sdv_TmpPDU_ValBl3
		max	wrot_max_TmpPDU_ValBl3
	wrot_V_ValBl3	min	wrot_min_V_ValBl3

		avg	wrot_avg_V_ValBl3
		sdv	wrot_sdv_V_ValBl3
		max	wrot_max_V_ValBl3
	wrot_TmpPwrSply_ValBl3	min	wrot_min_TmpPwrSply_ValBl3
		avg	wrot_avg_TmpPwrSply_ValBl3
		sdv	wrot_sdv_TmpPwrSply_ValBl3
		max	wrot_max_TmpPwrSply_ValBl3
WGEN	wgen_GnTmp_phsA	min	wgen_min_GnTmp_phsA
		avg	wgen_avg_GnTmp_phsA
		sdv	wgen_sdv_GnTmp_phsA
		max	wgen_max_GnTmp_phsA
	wgen_GnTmp_phsB	min	wgen_min_GnTmp_phsB
		avg	wgen_avg_GnTmp_phsB
		sdv	wgen_sdv_GnTmp_phsB
		max	wgen_max_GnTmp_phsB
	wgen_Spd	min	wgen_min_Spd
		avg	wgen_avg_Spd
		sdv	wgen_sdv_Spd
		max	wgen_max_Spd
	wgen_RtrSpd_WP2035	min	wgen_min_RtrSpd_WP2035
		avg	wgen_avg_RtrSpd_WP2035
		sdv	wgen_sdv_RtrSpd_WP2035
		max	wgen_max_RtrSpd_WP2035
	wgen_GnTmp_phsC	min	wgen_min_GnTmp_phsC
		avg	wgen_avg_GnTmp_phsC
		sdv	wgen_sdv_GnTmp_phsC
		max	wgen_max_GnTmp_phsC
	wgen_RtrSpd_IGR	min	wgen_min_RtrSpd_IGR
		avg	wgen_avg_RtrSpd_IGR
		sdv	wgen_sdv_RtrSpd_IGR
		max	wgen_max_RtrSpd_IGR
WNAC	wnac_WVaneDir1	min	wnac_min_WVaneDir1
		avg	wnac_avg_WVaneDir1
		sdv	wnac_sdv_WVaneDir1
		max	wnac_max_WVaneDir1
	wnac_WSpd2	min	wnac_min_WSpd2
		avg	wnac_avg_WSpd2
		sdv	wnac_sdv_WSpd2
		max	wnac_max_WSpd2
	wnac_Dir	min	wnac_min_Dir
		avg	wnac_avg_Dir
		sdv	wnac_sdv_Dir
		max	wnac_max_Dir
	wnac_Wdir1	min	wnac_min_Wdir1
		avg	wnac_avg_Wdir1
		sdv	wnac_sdv_Wdir1
		max	wnac_max_Wdir1
	wnac_WVaneDir2	min	wnac_min_WVaneDir2
		avg	wnac_avg_WVaneDir2
		sdv	wnac_sdv_WVaneDir2
		max	wnac_max_WVaneDir2
	wnac_WSpd1	min	wnac_min_WSpd1
		avg	wnac_avg_WSpd1
		sdv	wnac_sdv_WSpd1

		max	wnac_max_WSpd1
	wnac_Wdir2	min	wnac_min_Wdir2
		avg	wnac_avg_Wdir2
		sdv	wnac_sdv_Wdir2
		max	wnac_max_Wdir2
	wnac_ExlTmp	min	wnac_min_ExlTmp
		avg	wnac_avg_ExlTmp
		sdv	wnac_sdv_ExlTmp
		max	wnac_max_ExlTmp
	wnac_NacTmp	min	wnac_min_NacTmp
		avg	wnac_avg_NacTmp
		sdv	wnac_sdv_NacTmp
		max	wnac_max_NacTmp
WCNV	wcnv_InvTmp_ClWtrFwd	min	wcnv_min_InvTmp_ClWtrFwd
		avg	wcnv_avg_InvTmp_ClWtrFwd
		sdv	wcnv_sdv_InvTmp_ClWtrFwd
		max	wcnv_max_InvTmp_ClWtrFwd
	wcnv_IGBTTmp	min	wcnv_min_IGBTTmp
		avg	wcnv_avg_IGBTTmp
		sdv	wcnv_sdv_IGBTTmp
		max	wcnv_max_IGBTTmp
	wcnv_InvTmp_ClWtrRet	min	wcnv_min_InvTmp_ClWtrRet
		avg	wcnv_avg_InvTmp_ClWtrRet
		sdv	wcnv_sdv_InvTmp_ClWtrRet
		max	wcnv_max_InvTmp_ClWtrRet
	wcnv_HtSnkTmp	min	wcnv_min_HtSnkTmp
		avg	wcnv_avg_HtSnkTmp
		sdv	wcnv_sdv_HtSnkTmp
		max	wcnv_max_HtSnkTmp

Table 2. Complete variable list divided into systems and variable groups. Each variable group contains four statistical indicators, min/max/sdv/avg calculated from higher frequency sensors and summarized every 5 minutes.

3 Description of the alarms

Table 3 contains the list of alarms and warnings, which are mixed together in the database. Therefore we will use the name 'alarm' to refer to the set of alarms and warnings. The alarms are tagged by a numerical identifier (ID). There is a total of 369 different alarms. Each alarm is associated with a main system (SYS) and a subsystem in the system (SUBS). Moreover, there is a short description of each alarm (DESC).

ID	SYS	SUBS	DESC
0	Turbine	Control Cabinet	System OK
5	Turbine	Control Cabinet	Vibration
7	Turbine	Control Cabinet	Turbine is serviced
9	Turbine	Control Cabinet	Remote stop
13	Turbine	Control Cabinet	Manual stop
16	Turbine	Control Cabinet	Emer.stop cont.panel
23	Turbine	Control Cabinet	Repeating error
30	Nacelle	Control Cabinet	Nacelle temp.
31	Nacelle	Control Cabinet	Nacelle temp. stop
41	Turbine	Control Cabinet	UPS battery low
45	Turbine	Power Cabinet	Main ctrl. Supply
55	Turbine	Control Cabinet	Main ctrl.man.reboot
66	Turbine	Control Cabinet	Fire alarm
93	Turbine	Control Cabinet	Service hatch
100	Turbine	Control Cabinet	Repeated grid error

102	Turbine	Control Cabinet	Phase drop
103	Turbine	Control Cabinet	Vector surge
110	Turbine	Control Cabinet	Voltage high
111	Turbine	Control Cabinet	Voltage low
113	Transformer	Transformer	Trafo overtemp.
114	Transformer	Transformer	Trafo temp. stop
120	Turbine	Control Cabinet	Frequency high
121	Turbine	Control Cabinet	Frequency low
128	Turbine	Control Cabinet	Transient grid error
130	Turbine	Control Cabinet	L1-L2-L3 120
134	Turbine	Control Cabinet	Critical frequency
154	Transformer	Transformer	Trafo min. temp.
155	Transformer	Transformer	Trafo min temp. stop
200	Nacelle	Control Cabinet	Outdoor temp. low
201	Nacelle	Control Cabinet	Outdoor temp. high
202	Turbine	Control Cabinet	Wind < power
203	Turbine	Control Cabinet	Wind > power
205	Tower	Control Cabinet	Tower resonance time
206	Rotor	Control Cabinet	Ice warning
220	Turbine	Roof	Diff WindSpeedSens>SHH
221	Turbine	Roof	W.direc.nonidentical
226	Turbine	Roof	WindSpeedSensorsDefect
230	Turbine	Roof	WindSpeedSens1 defect
231	Turbine	Roof	WindSpeedSens2 defect
233	Turbine	Control Cabinet	WindSpeed<StartCond
235	Turbine	Control Cabinet	Wind vanes defect
236	Turbine	Control Cabinet	Out.temp low stop
237	Turbine	Control Cabinet	Out.temp hi. Stop
238	Turbine	Control Cabinet	Anemo.test rpm high
239	Turbine	Control Cabinet	Anemo.test rpm low
241	Turbine	Control Cabinet	Weather sensor com.
243	Turbine	Control Cabinet	Light.prot. term.box
250	Turbine	Control Cabinet	WindSpeed > SH
251	Turbine	Control Cabinet	WindSpeed > SHH
260	Turbine	Control Cabinet	Wind vane 1 defect
261	Turbine	Control Cabinet	Wind vane 2 defect
265	Turbine	Control Cabinet	Weather #1 Com.
266	Turbine	Control Cabinet	Weather #1
267	Turbine	Control Cabinet	Weather #1 Iced
320	Turbine	Control Cabinet	WP2035 (R) overspeed
346	Turbine	Control Cabinet	(G)oversp. operation
347	Turbine	Control Cabinet	Max. service (G)rpm
348	Turbine	Control Cabinet	WP2035 com. timeout
349	Turbine	Control Cabinet	WP2035 com.pack.err
350	Turbine	Control Cabinet	WP2035 <> rotor rpm
414	Transmission	Brake	MBS Not Open
415	Transmission	Brake	MBS Pads Wear
416	Transmission	Brake	MBS Pads Wear Warn
431	Generator	Generator	BP50 GenSpeedRed<min
474	Turbine	Control Cabinet	(B) air press. low
500	Generator	Generator	Gen Repeat Error
530	Turbine	Control Cabinet	ActivePower > SHH
531	Generator	Generator	Gen TempCoil L1 > SH
533	Generator	Generator	Gen TempCoil L2 > SH
535	Generator	Generator	Gen TempCoil L3 > SH

554	Generator	Generator	Gen WearBrush Warn
562	Generator	Generator	Gen TempCoolWatRet>SHH
601	Turbine	Control Cabinet	Current asymmetry
613	Transformer	Transformer	Trafo oil press.high
614	Transformer	Transformer	Trafo oil press.stop
640	Rotor	Hub	Lightningprot.hub
663	Transformer	Transformer	Transformer leaking
700	Yaw	Yaw	Error by yawing
701	Yaw	Yaw	Rep. error by yawing
708	Yaw	Yaw	Twisted CCW
709	Yaw	Yaw	Twisted CW
715	Yaw	Yaw	Cable autounwind
719	Yaw	Yaw	(H)yaw(M)therm.relay
730	Yaw	Yaw	Yaw sensor defect
731	Yaw	Yaw	Yaw(P) starting rate
732	Yaw	Yaw	Manual yaw active
733	Yaw	Yaw	Yaw(P) oper.time>max
734	Turbine	Control Cabinet	Nacel.pos<>wind vane
742	Yaw	Yaw	Yaw sensor A/B
743	Yaw	Yaw	Yaw thermal relay
760	Yaw	Yaw	Yaw misalignment
770	Yaw	Yaw	Yaw error inverter 1
771	Yaw	Yaw	Yaw error inverter 2
772	Yaw	Yaw	Yaw error inverter 3
773	Yaw	Yaw	Yaw error inverter 4
775	Yaw	Yaw	LubYaw Rim Time-out
776	Yaw	Yaw	LubYawBear Time-out
779	Yaw	Yaw	LubYaw Rim GreaseEmpty
780	Yaw	Yaw	LubYawBearGreaseEmpty
904	Generator	Generator	Gen FuseTripCoolWatPp
907	Nacelle	Control Cabinet	Therm. nacelle fan
916	Transmission	Gearbox	MGB Temp CoolWater > SHH
944	Transmission	Main Bearing	MGB PressSwitchCoolWat
963	Transmission	Gearbox	MGB RepeatCoolWatError
964	Transmission	Main Bearing	MGB FuseTrip OilPump
1016	Rotor	Pitch	Ethcan pitch com
1017	Rotor	Pitch	CANopen pitch com
1018	Rotor	Pitch	Ethcan inv. Com
1019	Rotor	Pitch	CANopen inv. Com
1020	Rotor	Pitch	Pitch 1 CANopen
1021	Rotor	Pitch	Pitch 2 CANopen
1022	Rotor	Pitch	Pitch 3 CANopen
1023	Rotor	Pitch	Inverter CANopen
1024	Rotor	Pitch	Ethcan pitch 1 emcy.
1025	Rotor	Pitch	Ethcan pitch 2 emcy.
1026	Rotor	Pitch	Ethcan pitch 3 emcy.
1027	Rotor	Pitch	Ethcan inv. emcy.
1028	Rotor	Pitch	Ethnet pitch receive
1029	Rotor	Pitch	Ethnet inv. receive
1030	Rotor	Pitch	Ethnet pitch send
1031	Rotor	Pitch	Ethnet inv. send
1113	Rotor	Pitch	PLU DeltaPitchAngle>SHH
1207	Transmission	Main Bearing	MBS FuseTrip HydOilPp
1210	Transmission	Main Bearing	M.bear.Level Oil < min
1213	Transmission	Main Bearing	MMBSOilPp RunTime>max

1215	Transmission	Main Bearing	MBSOilPpStartingRate
1224	Transmission	Main Bearing	MBSPress HydOil < SL
1271	Transmission	Gearbox	MGB FuseTripCoolWatPp
1272	Transmission	Main Bearing	M.bear.PressOil IN>SH
1273	Transmission	Main Bearing	M.bear. Error Pressure
1280	Transmission	Main Bearing	M.bear. Press Oil IN < SL
1306	Transmission	Main Bearing	MGB PressOil In < SL
1329	Transmission	Main Bearing	MGB FuseTripOilHeater
1359	Transmission	Main Bearing	M.bear. Temp 1 > SHH
1360	Transmission	Main Bearing	M.bear. Temp 1 > SH
1361	Transmission	Main Bearing	M.bear. Temp 1 < SL
1362	Transmission	Main Bearing	M.bear. Temp 1 < SLL
1363	Transmission	Main Bearing	M.bear. Temp 2 > SHH
1364	Transmission	Main Bearing	M.bear. Temp 2 > SH
1365	Transmission	Main Bearing	M.bear. Temp 2 < SL
1366	Transmission	Main Bearing	M.bear. Temp 2 < SLL
1367	Transmission	Gearbox	MGB TempOilSump > SHH
1368	Transmission	Gearbox	MGB Temp OilSump > SH
1369	Transmission	Gearbox	MGB Temp OilSump < SL
1370	Transmission	Gearbox	MGB Temp OilSump < SLL
1371	Transmission	Gearbox	MGB Repeat Temp Error
1372	Transmission	Gearbox	MGB TempBear151 > SHH
1373	Transmission	Gearbox	MGB TempBear451 > SHH
1374	Transmission	Gearbox	MGB TempBear150 > SHH
1375	Transmission	Gearbox	MGB TempBear450 > SHH
1376	Transmission	Gearbox	MGB TempBear152 > SHH
1377	Transmission	Gearbox	MGB TempBear452 > SHH
1378	Transmission	Gearbox	MGB FilterOil Warning
1379	Transmission	Gearbox	1379: MGB Filter Oil Stop
1380	Transmission	Main Bearing	M.bear.FuseTrip OilPp
1381	Transmission	Main Bearing	M.bear. PressOilPp<SL
1382	Transmission	Main Bearing	M.bear. FilterOilStop
1392	Transmission	Main Bearing	M.bear. FilterOilWarn
1402	Converter	Converter	Freq. conv. warning
1404	Converter	Converter	Freq.conv. emer.stop
1406	Converter	Converter	Freq.conv.grid error
1409	Converter	Converter	Freq. conv. error
1411	Converter	Converter	Freq.conv.overspeed
1412	Converter	Converter	Timeout Freq. conv.
1415	Converter	Converter	Freq.conv. <> sync.
1498	Converter	Converter	Freq.con. heat oper.
1544	Transmission	Gearbox	PT100 defective
1588	Turbine	Control Cabinet	4-20mA signal defect
1595	Turbine	Control Cabinet	Selftest
1667	Converter	Converter	Freq. conv. communi.
1668	Converter	Converter	Freq. conv. error A
1669	Converter	Converter	Freq. conv. error B
1671	Converter	Converter	F.conv. MCCB tripped
1672	Converter	Converter	F.conv. MCCB open
1673	Converter	Converter	Freq.conv.UPS defect
1674	Converter	Converter	F.conv.power high
1684	Converter	Converter	F.conv. trans. grid
1685	Turbine	Control Cabinet	Service active LVU
1689	Converter	Converter	Timeout grid connect
1702	Transmission	Main Bearing	M.bear. TempRepeatErr

1715	Turbine	Control Cabinet	4-20mA signal stop
1743	Turbine	Control Cabinet	Emer. stop 1 nacelle
1744	Turbine	Control Cabinet	Emer. stop 2 nacelle
1768	Turbine	Control Cabinet	Service mode
1769	Turbine	Control Cabinet	Main ctrl.m.shutdown
1791	Turbine	Control Cabinet	[P] reduced temp.
1792	Turbine	Control Cabinet	[P] reduced conv.
1793	Turbine	Control Cabinet	[P] reduced EU
1813	Transmission	Brake	MBS Pads Wear Stop
1814	Transmission	Hydraulic System	(H)(B)press hi. Test
1815	Transmission	Hydraulic System	(H)(B)press lo. Test
1816	Transmission	Hydraulic System	(H)(B)press Test
1817	Transmission	Hydraulic System	(B) Press. Time out
1818	Transmission	Hydraulic System	(B) Press. Delayed
1819	Transmission	Hydraulic System	(B) Press. Deviation
1820	Transmission	Hydraulic System	(B)Press.Limit valve
1821	Transmission	Hydraulic System	(B)Press.Complete
1822	Transmission	Hydraulic System	(B) Press. Undelayed
1823	Transmission	Hydraulic System	(B)Time out test
1907	Rotor	Pitch	LubPitch Rim Time-out
1919	Rotor	Pitch	Pitch 1 too slow
1920	Rotor	Pitch	Pitch 2 too slow
1921	Rotor	Pitch	Pitch 3 too slow
1925	Rotor	Pitch	Pitch 1 pos. FB
1926	Rotor	Pitch	Pitch 2 pos. FB
1927	Rotor	Pitch	Pitch 3 pos. FB
1928	Rotor	Pitch	Capacitor volt. low
1929	Rotor	Pitch	Capacitor volt.high
1930	Rotor	Pitch	Capacitor capac. low
1934	Rotor	Pitch	LubPitchRimGreaseEmpty
1942	Rotor	Rotor	Service active hub
1944	Rotor	Blade	MainSwitchOff blade1
1945	Rotor	Blade	MainSwitchOff blade2
1946	Rotor	Blade	MainSwitchOff blade3
1947	Rotor	Pitch	PLU com. error
1948	Rotor	Pitch	PLU PBU Voltage4<SLL
1949	Rotor	Pitch	PLU safety sys. rel.
1950	Rotor	Pitch	PLU run away
1951	Rotor	Pitch	PLU EndStop100 PS2
1952	Rotor	Pitch	PLU PCO Error
1954	Rotor	Pitch	PLU safety run error
1955	Rotor	Pitch	PLU PCH Error
1956	Rotor	Pitch	PLU PCH Warning
1957	Rotor	Pitch	PLU HMI serv. switch
1958	Rotor	Pitch	PLU1 EndStop -5 PS2
1959	Rotor	Pitch	PLU EndStop100 PS1
1960	Rotor	Pitch	PLU Brake Not Open
1961	Rotor	Pitch	PLU blade fixed
1963	Rotor	Pitch	PLU PBU Voltage4>SHH
1964	Rotor	Pitch	PLU1 PBUDeltaVolt>SHH
1965	Rotor	Pitch	PLU2 PBUDeltaVolt>SHH
1966	Rotor	Pitch	PLU3 PBUDeltaVolt>SHH
1967	Rotor	Pitch	PLU PBU Temp>SHH
1968	Rotor	Pitch	PLU PCOTempHeatSink>SHH
1969	Rotor	Pitch	PLU PDU Temp>SHH

1970	Rotor	Pitch	PLU PCH Temp>SHH
1986	Rotor	Pitch	PLU PCO Temp>SHH
1987	Rotor	Pitch	PLU BladeAngleDelta>SHH
1994	Rotor	Pitch	PLU Pitch Angle>SH
2027	Generator	Generator	Gen PressSwitchCoolWat
2028	Generator	Generator	Gen RepeatCoolWatError
2029	Generator	Generator	LubGen GreaseEmpty
2034	Generator	Generator	LubGen Time-out
2035	Generator	Generator	GenTempBear DE >SH<SL
2037	Generator	Generator	GenTempBearNDE >SH<SL
2040	Generator	Generator	Gen FuseTrip Heater
2046	Generator	Generator	Service act tm. Box
2047	Generator	Generator	Gen WearBrush Stop
2048	Generator	Generator	Gen TempCoil L1 < SL
2049	Generator	Generator	Gen TempCoil L2 < SL
2050	Generator	Generator	Gen TempCoil L3 < SL
2058	Generator	Generator	Gen TempCoil L1 > SHH
2059	Generator	Generator	Gen TempCoil L2 > SHH
2060	Generator	Generator	Gen TempCoil L3 > SHH
2061	Generator	Generator	Gen TempCoil L1 < SLL
2062	Generator	Generator	Gen TempCoil L2 < SLL
2063	Generator	Generator	Gen TempCoil L3 < SLL
2064	Generator	Generator	Gen TempBearNDE > SHH
2065	Generator	Generator	Gen TempBearNDE < SLL
2066	Generator	Generator	Gen TempBear DE > SHH
2067	Generator	Generator	Gen TempBear DE < SLL
2120	Turbine	Control Cabinet	Turbine maintenance
2135	Turbine	Control Cabinet	Check alarm connect.
2142	Transmission	Main Bearing	M.bear.FuseTripOilHeat
2154	Turbine	Power Cabinet	Emer.stop med.volt
2166	Turbine	Power Cabinet	Terminal box temp.
2167	Turbine	Power Cabinet	LV unit temp. high
2168	Turbine	Power Cabinet	LV unit th.rel.
2169	Turbine	Power Cabinet	MV unit MCCB open
2170	Turbine	Power Cabinet	MV unit MCCB releas.
2171	Turbine	Power Cabinet	MV unit PE-br.1 clos
2172	Turbine	Power Cabinet	MV unit PE-br.2 clos
2173	Turbine	Control Cabinet	Safety sys activated
2174	Turbine	Control Cabinet	Safety sys def.
2225	Turbine	Control Cabinet	UPS Comm. Lost
2235	Turbine	Control Cabinet	Obstr.light grid
2236	Turbine	Roof	Obstr.light one lamp
2237	Turbine	Roof	Obstr.light two lamp
2238	Turbine	Control Cabinet	Acc.nacelle Y warn.
2239	Turbine	Control Cabinet	Acc.nacelle Z warn.
2240	Turbine	Control Cabinet	Acc.nacelle Y stop
2241	Turbine	Control Cabinet	Acc.nacelle Z stop
2242	Tower	Tower	Tower torsion warn.
2243	Tower	Tower	Tower torsion stop
2244	Turbine	Control Cabinet	Acc.nacel.filt.Y war
2245	Turbine	Control Cabinet	Acc.nacel.filt.Z war
2246	Turbine	Control Cabinet	Acc.nacel.filt.Y stp
2247	Turbine	Control Cabinet	Acc.nacel.filt.Z stp
2248	Turbine	Control Cabinet	ACS watchdog
2249	Turbine	Control Cabinet	ACS error

2250	Turbine	Control Cabinet	Resid.curr.guard war
2251	Turbine	Control Cabinet	Resid.curr.guard stp
2252	Turbine	Control Cabinet	CMS watchdog
2253	Turbine	Control Cabinet	CMS warning
2254	Turbine	Control Cabinet	CMS stop
2269	Turbine	Control Cabinet	WP4086#1 acc. Alarm
2270	Turbine	Control Cabinet	WP4086#1 acc. warn.
2273	Turbine	Control Cabinet	WP4086#1 lev.1 alarm
2274	Turbine	Control Cabinet	WP4086#1 lev.1 warn.
2275	Turbine	Control Cabinet	WP4086#1 lev.2 alarm
2276	Turbine	Control Cabinet	WP4086#1 lev.2 warn.
2277	Turbine	Control Cabinet	WP4086#1 lev.3 alarm
2278	Turbine	Control Cabinet	WP4086#1 lev.3 warn.
2279	Turbine	Control Cabinet	WP4086 comm. Error
2285	Turbine	Control Cabinet	App. system err.
2290	Turbine	Control Cabinet	WP4086#1 log ready.
2291	Turbine	Control Cabinet	WP4086#1 Intern Err.
2300	Transmission	Gearbox	MGB TempBear151 > SH
2301	Transmission	Gearbox	MGB TempBear451 > SH
2302	Transmission	Gearbox	MGB TempBear150 > SH
2303	Transmission	Gearbox	MGB TempBear450 > SH
2304	Transmission	Gearbox	MGB TempBear152 > SH
2305	Transmission	Gearbox	MGB TempBear452 > SH
2306	Transmission	Gearbox	MGB Temp ErrTimeLimit
2423	Turbine	Control Cabinet	Fire protect warn. 1
2424	Turbine	Control Cabinet	Fire protect warn. 2
2425	Turbine	Control Cabinet	Fire protect warn. 3
2426	Turbine	Control Cabinet	Fire protect warn. 4
2427	Turbine	Control Cabinet	Fire protect warn. 5
2476	Turbine	Control Cabinet	App. Not configured
2491	Turbine	Control Cabinet	Repeating alarm
2701	Turbine	Control Cabinet	No PMS available
2703	Turbine	Control Cabinet	PMC stop
3025	Turbine	Control Cabinet	Stop by SCADA
3108	Turbine	Control Cabinet	TSO Stop
3109	Turbine	Control Cabinet	APC Local Mode
3110	Turbine	Control Cabinet	APC Setpoint !
3111	Turbine	Control Cabinet	RPC Local Mode
3112	Turbine	Control Cabinet	RPC Setpoint
3113	Turbine	Control Cabinet	P > P Sp
3119	Turbine	Control Cabinet	GCA Grid
3120	Turbine	Control Cabinet	GCA Grid Con. Inhibit
3121	Turbine	Control Cabinet	TSO Reconnect. Block
3122	Turbine	Control Cabinet	GCA Manual Stop
3123	Turbine	Control Cabinet	GCA Stop
5000	Turbine	Control Cabinet	WTG System OK
5131	Turbine	Control Cabinet	Wrong App 50Hz 60 Hz
5132	Turbine	Control Cabinet	Communication TEST
5135	Turbine	Control Cabinet	App. Not configured
5194	Turbine	Control Cabinet	Customer ON
5195	Turbine	Control Cabinet	Customer OFF
5196	Turbine	Control Cabinet	Service ON
5197	Turbine	Control Cabinet	Service OFF
5198	Turbine	Control Cabinet	Maintenance ON
5199	Turbine	Control Cabinet	Maintenance OFF

5213	Turbine	Control Cabinet	PLU PCO warning
5235	Rotor	Rotor	Rotor Speed > SH
5256	Rotor	Pitch	PBU Error
5257	Rotor	Pitch	PBU Warning
5345	Turbine	Control Cabinet	NacTowerUPSLightWarn
5361	Rotor	Blade	BladeCtrl Error
5362	Rotor	Blade	BladeCtrl Warning
5363	Rotor	Blade	BladeDamage
5430	Turbine	Control Cabinet	GridFQ<StartCondition
5473	Turbine	Control Cabinet	LVU LightingProtWarn1
5474	Turbine	Control Cabinet	LVU LightingProtWarn2
5494	Turbine	Control Cabinet	LVU ExternalStop
5495	Turbine	Control Cabinet	External warning
5496	Turbine	Control Cabinet	TB External stop 1
5497	Turbine	Control Cabinet	TB ExternalStop 2
5498	Turbine	Control Cabinet	TB ExternalStop 3
5499	Turbine	Control Cabinet	TB ExternalStop 4
5705	Generator	Generator	Gen Speed > SHH
5706	Generator	Generator	Gen <> RotSafSys > SH
5707	Yaw	Yaw	Gen <> RotIGR > SH
5815	Yaw	Yaw	Yaw Inv Error Repeat
5930	Yaw	Yaw	LubYaw GreaseEmpty
5931	Yaw	Yaw	LubYaw Pressure Error
5932	Yaw	Yaw	LubYaw Error

Table 3. Complete list of alarms provided by the manufacturer. Each alarm is identified by a numeric ID. Note that the ID numbers are all integers, ordered from lowest to highest, but not consecutive.

4 Options of the function *get_turbine_data*

This function is the one used to obtain the wind turbine data for specific WTs, period of time, and type of pre-processing, together with the selected alarms.

The function is called as follows:

```
[error,data,msg]=get_turbine_data(compressed_file_name, alarm_id\_list,
    frequency_seconds, combine_func, one_hot_encoding, threads, verbose)
```

Where the parameter frequency_seconds allows merging the data in blocks according to the selected frequency (in seconds), to decrease the sampling rate if needed.

The pre-processing (filtering of outliers) is controlled with the combine_functions, which has the following possible values:

- "mean": Average of aggregated data inside the block
- "median": Median of aggregated data inside the block
- "max": Max of aggregated data inside the block"
- "min": Min of aggregated data inside the block"
- "filtered_3sdv_mean": Within each aggregated block of data, a filtering process removes all values outside the range mean$\pm$3sdv. The mean of the remaining values is then calculated.
- "filtered_3sdv_median": Within each aggregated block of data, a filtering process removes all values outside the range mean$\pm$3sdv. The median of the remaining values is then calculated.
- "filtered_mad_mean": Within each aggregated block of data, a filtering process removes all values outside the range median$\pm$2MAD. The mean of the remaining values is then calculated.
- "filtered_mad_median": Within each aggregated block of data, a filtering process removes all values outside the range median$\pm$2MAD. The median of the remaining values is then calculated.

Note that in the first four options the outliers are not filtered, while in the last four, the values are filtered using two different criteria, and the mean or the median values are calculated after this pre-processing.

Finally, one_hot_encoding is used to determine how the alarm information is coded. If TRUE, a column will be generated for each alarm starting with "alarm_" and the alarm id with 0 (not active) and 1 (active). If FALSE, a single column will be generated with the name alarms_active including a comma-separated list of active alarms.
